# A structural brain network of genetic vulnerability to psychiatric illness

**DOI:** 10.1038/s41380-020-0723-7

**Published:** 2020-05-06

**Authors:** Maxime Taquet, Stephen M. Smith, Anna K. Prohl, Jurriaan M. Peters, Simon K. Warfield, Benoit Scherrer, Paul J. Harrison

**Affiliations:** 1 Department of Psychiatry, University of Oxford, Oxford, UK; 2 Computational Radiology Laboratory, Boston Children’s Hospital, Harvard Medical School, Boston, MA USA; 3 Wellcome Centre for Integrative Neuroimaging (WIN FMRIB), University of Oxford, Oxford, UK; 4 Division of Epilepsy and Clinical Neurophysiology, Department of Neurology, Boston Children’s Hospital, Harvard Medical School, Boston, MA USA; 5 Oxford Health NHS Foundation Trust, Oxford, UK

**Keywords:** Psychiatric disorders, Predictive markers

## Abstract

Psychiatry is undergoing a paradigm shift from the acceptance of distinct diagnoses to a representation of psychiatric illness that crosses diagnostic boundaries. How this transition is supported by a shared neurobiology remains largely unknown. In this study, we first identify single nucleotide polymorphisms (SNPs) associated with psychiatric disorders based on 136 genome-wide association studies. We then conduct a joint analysis of these SNPs and brain structural connectomes in 678 healthy children in the PING study. We discovered a strong, robust, and transdiagnostic mode of genome–connectome covariation which is positively and specifically correlated with genetic risk for psychiatric illness at the level of individual SNPs. Similarly, this mode is also significantly positively correlated with polygenic risk scores for schizophrenia, alcohol use disorder, major depressive disorder, a combined bipolar disorder-schizophrenia phenotype, and a broader cross-disorder phenotype, and significantly negatively correlated with a polygenic risk score for educational attainment. The resulting “vulnerability network” is shown to mediate the influence of genetic risks onto behaviors related to psychiatric vulnerability (e.g., marijuana, alcohol, and caffeine misuse, perceived stress, and impulsive behavior). Its anatomy overlaps with the default-mode network, with a network of cognitive control, and with the occipital cortex. These findings suggest that the brain vulnerability network represents an endophenotype funneling genetic risks for various psychiatric illnesses through a common neurobiological root. It may form part of the neural underpinning of the well-recognized but poorly explained overlap and comorbidity between psychiatric disorders.

## Introduction

The representation of mental illness along dimensions that cross diagnostic boundaries is progressively replacing the acceptance of distinct psychiatric diagnoses [1]. This paradigm shift is supported by the high comorbidity between disorders [2, 3], their clinical overlap [4], and their shared heritability [5]. This transition holds promise to identify more reliable biomarkers [6] and develop novel treatments [7] for psychiatric diseases. A crucial step in this endeavor is to unravel shared biological mechanisms underpinning different psychiatric illnesses. One hypothesis is that psychiatric illness reflects largely common disruptions in brain circuits encoding core dimensions of cognition and affect [8]. In support of this hypothesis, case-control neuroimaging studies have identified brain circuit disruptions shared across psychiatric disorders [9–11] and a recent study has shown associations between white matter properties and a transdiagnostic behavioral measurement of psychopathology [12]. However, the etiology and interpretation of these associations between neuroimaging findings and diagnostic categories or behaviors remain unclear. They may reflect shared genetic risks, environmental risks, consequences of the disease or behaviors, attempted adaptive responses to illness, or a combination thereof [13]. Understanding causal mechanisms of cross-disorder disease connectomics therefore calls for study designs that go beyond comparing patients with healthy controls [13]. For specific disorders, a few studies have shown that connectomic alterations are present in at-risk individuals (i.e., individuals with a high clinical risk for the disorder, or with an affected parent) providing initial evidence that some connectomic changes may precede the onset of illness [14, 15]. Whether these findings form part of a more general overarching vulnerability to psychiatric illness across diagnostic categories and whether this vulnerability can be imputed to genetic factors remain unknown.

To begin to address this question, we conducted an integrated analysis of structural connectomes (i.e., comprehensive maps of the white matter connections in the brain) and single nucleotide polymorphisms (SNPs) in healthy children. Our analysis focused on SNPs with an established risk for psychiatric illness or for phenotypes related to brain connectivity (i.e., neurological diseases, behavior-cognition, and brain morphology/function). The aim of this analysis was to assess whether genetic risk factors for psychiatric illness are associated with specific patterns of brain connectivity. We used data from 678 children and adolescents in the Pediatric Imaging, Neurocognition, and Genetics (PING) cohort—one of the largest neuroimaging and genetic datasets in healthy children [16]. Importantly, the use of a healthy population factors out the impact of illness state, its sequelae, and treatments, while the use of a pediatric population limits the impact of different environmental exposures on the brain [17]. We used canonical correlation analysis (CCA), which has been applied previously to identify a rich relationship between functional connectomes, demographics, and behavior [18]. The primary input to CCA were data for 1877 SNPs and whole-brain structural connectomes obtained from diffusion MRI (dMRI). CCA seeks modes of population covariation that goes beyond testing whether a predetermined linear combination of SNPs for a specific disorder correlates with a brain imaging phenotype. To complement this approach, we also explored relationships between psychiatric polygenic risk scores (PRS) and the structural connectome.

## Methods and materials

### Participants

All data for this study come from the PING dataset. Recruitment and exclusion criteria are described elsewhere [16]. Participants and their parents gave their written informed consent or assent to participate in study procedures. The Institutional Review Board of data collection sites approved the study. All analyses were conducted on all participants who, as of April 2017, had a T1- and T2-weighted MRI, a full set of dMRI acquired with the PING research protocol, and genotype data. This resulted in a total of 678 participants (336 female, 342 male, mean [SD] age: 12.8 years [4.87 years]; see demographics details in Supplementary Table 1).

### Genetic data

The genetic data acquisition (of 550,000 SNPs) and quality control is described elsewhere [19]. SNPs of interest were selected as follows (see also Supplementary Figs. 1, 2 and Supplementary Information). Phenotypes of interest (Supplementary Table 2) were first identified from the NHGRI-EBI genome-wide association studies (GWAS) Catalog [20], which references all major GWAS to date. SNPs associated with a phenotype of interest at genome-wide significance (*p* < 5 × 10^−8^), as reported in the catalog, were identified. When an identified SNP was not part of the PING data, proxies in linkage disequilibrium with that SNP (with *R*^2^ > 0.8) were selected instead, in line with standard practice [21]. This process resulted in data for 1877 SNPs based on 136 GWAS for each of the 678 participants. The same process was repeated for SNPs associated with asthma and Type 2 diabetes; these phenotypes were used to test whether modes of covariation are specific to psychiatry or reflect a more general dimension of health. They were selected as archetypal examples of diseases with an established genetic basis and affecting mostly children and mostly adults, respectively.

### Social, emotional, and behavioral data

The PhenX questionnaire comprising social, emotional, and behavioral variables [16, 22] was administered to 117 participants of the PING cohort. This questionnaire was always acquired after the imaging data (mean [SD] delay: 15.6 months [7.3 months]; Supplementary Table 1). Only variables with over ten observations and with at least 5% of observations different from the rest (for dichotomous variables) were kept. Data for these variables were encoded so that higher values relate to increased psychiatric vulnerability.

### Structural connectomes

Details of the MRI acquisition are described elsewhere [16]. The MRI preprocessing is described in the supplementary information.

For each subject, a multi-tensor diffusion model was estimated from the preprocessed dMRI data [23]. This multi-tensor model accounts for crossing white matter fascicles and partial volumes of cerebrospinal fluid. The fractional anisotropy (FA) of each tensor represents the fascicle-specific FA (fFA), rather than the voxel-wise FA which is artifactually low in areas of crossing fascicles.

Whole-brain tractography was then performed using a multi-peak stochastic tractography algorithm. The streamlines were initiated at the white/gray matter interface [24] to increase the tractography reproducibility [25]. The seeding mask was constructed by computing the intersection between the white matter mask and a 2-voxel dilation of the gray matter mask. Fifteen streamlines were stochastically initiated in each seeding voxel. The streamlines were then propagated throughout the multi-tensor field by following, at each step, the tensor of the sub-voxel-interpolated multi-tensor model [26] best aligned with the current streamline trajectory incorporating both momentum strategy [27] and tensor deflection [28]. Streamlines were stopped when they (i) touched the gray matter, (ii) reached a curvature larger than 30°, or (iii) reached an fFA < 0.2.

The nodes of the structural connectome were defined by parcellation of the gray matter into 120 regions [29–31] (see Supplementary Information). Connectome matrices (symmetric 120 × 120 matrices) were obtained by computing for each pair of regions, the average fFA of streamlines connecting them [27]. Entries in these matrices are referred to as connection strengths.

### CCA modeling

Data were recorded as a 678 × 7140 connectomic data matrix containing all 7140 connection strengths for all subjects, and a 678 × 1877 genomic data matrix containing all 1877 SNPs for all subjects. The connectomic data matrix was adjusted for age, sex, scanner site, and genetic ancestry factors (GAFs) to avoid spurious correlations caused by these confounds (see below). To avoid overfitting in the CCA estimation, dimensionality reduction with principal component analysis (keeping 100 principal components as in [18]) was applied to the genomic and adjusted connectomic data matrices independently. The resulting two 678 × 100 dimensionality-reduced matrices were the inputs to CCA in Matlab 2015a.

CCA results in symmetric linear relationships between two sets of variables [32]. Here, each such relationship is a linear combination of SNPs called genetic canonical score and a linear combination of connection strengths called connectomic canonical score so that each individual is represented by two scores. Each linear relationship identified with CCA represents a mode of genome–connectome covariation in the sense that participants with a high genetic canonical score tend to have a high connectomic canonical score.

We also define canonical strengths which are properties of specific connections and SNPs and denote the extent to which these variables are correlated with the modes of covariation. Canonical genetic strengths are defined as the correlation between a SNP and the genetic canonical score and canonical connection strengths are defined as the correlation between a connection strength and the connectomic canonical score.

### Gene ontology

The genes that SNPs map to were recorded from the GWAS catalog. We used PANTHER-GO 14.0 [33] with Fisher’s exact test and Bonferroni correction to identify which biological processes, cellular components, and molecular functions were most enriched against 20,996 background human genes.

### Polygenic risk scores

The focus on SNPs of interest enables interpretation of modes of genome–connectome covariation in terms of etiological pathways. However, individual SNPs are poor predictors of current psychiatric phenotypes. PRS computed from summary statistics of GWAS incorporate information from the entire genome and are much more predictive at the expense of being less directly interpretable [34]. The two approaches thus complement one another. PRS were used in the present study to assess whether modes of genome–connectome covariation were related to increased genetic risks for different psychiatric phenotypes.

All phenotypes meeting the following criteria were selected for PRS analysis: (i) at least one GWAS has been conducted for that phenotype and its summary statistics made publicly available on the Psychiatric Genomics Consortium repository, (ii) a PRS has been calculated for that phenotype using the same summary statistics, (iii) the PRS explains at least 1% of the variance in that phenotype as validated within at least one external population. This resulted in PRS available for nine psychiatric phenotypes: major depressive disorder [35], bipolar disorder [36], schizophrenia [37], alcohol use disorder [38], autism spectrum disorders [39], eating disorder [40], attention-deficit hyperactivity disorder [41], a combined bipolar disorder and schizophrenia phenotype [42], and a broader cross-disorder phenotype [43]. We also added a PRS for educational attainment (which is known to predict well over 1% of the variance in this phenotype [44]) as this phenotype was also found to be (negatively) associated with the brain vulnerability network at the level of index SNPs.

PRS for each phenotype were calculated using PRSice 2.0 (which uses PLINK 1.9) [45]. The summary statistics of the original study (i.e., GWAS or meta-analysis thereof) were used as the base populations and the PING cohort was used as the target population. High-resolution *p*-thresholding is used by default in PRSice (this includes *p*-thresholds from 5 × 10^−8^ to 1.0 by steps of 5 × 10^−5^). It was used in this analysis, albeit with an upper bound set to the optimal threshold identified in the original study (since by definition SNPs not meeting this threshold do not contribute predictive power to the phenotype of interest).

### Statistical analysis

Statistical analyses were performed in R 3.4.3. Brain connection strengths (*C*) were adjusted for age, sex, scanner, and GAFs [16, 19] by linear regression:

$$C = C_0 + \beta _{{\mathrm{age}}}{\mathrm{age}} + \beta _{{\mathrm{sex}}}{\mathrm{sex}} + \beta _{{\mathrm{scanner}}}{\mathrm{scanner}} + \beta _{\mathrm{GAF}}{\mathrm{GAF}}.$$

The data-driven selection of covariates is described in the Supplementary Information.

To assess whether the modes of covariation were statistically significant, permutation tests with 10,000 permutations were applied. At each permutation, the subject IDs of one input matrix were randomly permuted relative to the other, CCA was applied to the result, and the maximum correlation coefficient achieved (in absolute value) was recorded. Comparison against this null distribution of maximum correlation coefficients therefore controls for multiple comparisons across modes of covariation.

The canonical genetic and connection strengths were also computed within each permutation resulting in *p* values for these variables. FDR-correction for multiple comparison using the Benjamini–Hochberg method [46] was applied to all canonical connection strengths together and all canonical genetic strengths within each phenotype.

Because CCA seeks the highest correlations in the data, it results in large correlations even under the null hypothesis. *P* values obtained from permutation tests account for this. However, to assess effect sizes, we computed the proportion of the variance in connectomes and SNPs explained by the modes of covariation and compared it with that explained under the null, following the same approach as in [18].

The correlation between the PRS for psychiatric phenotypes and the connectomic canonical score was calculated at each *p*-threshold using PRSice 2.0 [45]. GAFs were included as covariates in the analysis (even though the connectomic data matrix was already adjusted for these factors) to control for any residual influence of such factors at the polygenic level. This was achieved in PRSice 2.0 by subtracting from the *R*^2^ of the full regression model (including PRS and GAFs as independent variables), the *R*^2^ of the null model (in which only GAFs are included as independent variables). The two-tailed *p* value for the association between the PRS for a base phenotype and the connectomic canonical score was obtained using permutation tests (as implemented in PRSice 2.0), which correct for the multiple comparisons resulting from testing associations at different *p*-thresholds.

Social, emotional, and behavioral variables were adjusted for age and sex via linear regression for continuous variables,

$$Y = Y_0 + \beta _{{\mathrm{age}}}{\mathrm{age}} + \beta _{{\mathrm{sex}}}{\mathrm{sex}},$$

and via logistic regression for dichotomous variables,

$${\mathrm{logit}}\,P\left( Y \right) = L_0 + \beta _{{\mathrm{age}}}{\mathrm{age}} + \beta _{{\mathrm{sex}}}{\mathrm{sex}}.$$

To test the hypothesis that canonical brain networks are associated with these adjusted variables, correlation coefficients were first calculated between them and the connectomic canonical scores. The corresponding *p* values were collectively analyzed using a permutation-based combined probability test [47] with 1000 permutations. This test accounts for nonindependent *p* values (since some social, emotional, and behavioral variables are highly correlated). One-tailed *p* values were used as inputs to the combined probability test to guarantee that a significant result reflects a positive correlation between the connectomic canonical scores and behaviors related to psychiatric vulnerability (and not just any association between the mode of covariation and social, emotional, and behavioral variables, see Supplementary Information).

We applied mediation analysis (using the lavaan R package 0.6–3 [48]) to test whether significant correlations between connectomic canonical scores and social, emotional, and behavioral variables might reflect a mediation of the effect of genetic risks on behaviors through brain networks [49]. In this analysis, the genetic canonical score was the independent variable, social, emotional, and behavioral variables were dependent variables, and the connectomic canonical score was the mediator. The *p* values for the mediation of connectomic canonical scores on dependent variables were collectively analyzed using the same permutation-based combined probability test as above [47].

### Robustness analysis

Robustness of the results was evaluated by reproducing the analysis in six scenarios: (i) in two mutually exclusive subgroups randomly selected from the population, (ii) after restricting the subjects to a more homogenous population of European genetic ancestry, (iii) by adjusting SNPs and brain connections (rather than brain connections alone) to ancestry factors, (iv) after changing the number of principal components retained, (v) by restricting the input SNPs to specific subgroups of psychiatric phenotypes thereby testing whether canonical brain networks are truly transdiagnostic, (vi) after excluding participants exhibiting behaviors related to psychiatric vulnerability. Details of these analyses are given in the Supplementary Information.

## Results

Our analysis revealed five main findings. First, there was a single, highly significant, mode of genome–connectome covariation (correlation between genetic and connectomic canonical scores: *r* = 0.74, permutation test: *p* < 10^−4^ corrected for multiple comparisons across all modes estimated). It explains 6.43 times more of the variance in connectomes, and 3.33 times more of the variance in SNPs, than does the null distribution of CCA modes. All subsequent modes were non-significant (*p* > 0.1).

**Fig. 1:**
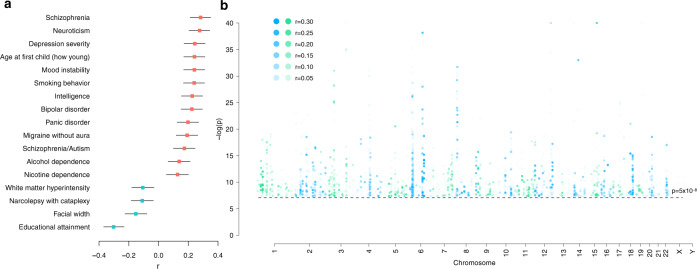
Distribution of SNPs and their correlation with the mode of population covariation. **a** Correlation between the top hit for each phenotype and the mode of population covariation showing that positive associations were found for SNPs associated with a range of psychiatric and cognitive-behavioral phenotypes. Error bars indicate 95% confidence intervals. **b** Genomic position of the 1877 SNPs selected for the analysis on the genome colored by their correlation (*r*) with the CCA mode. Only SNPs which were reported in the GWAS catalog to be significantly associated with phenotypes of interest (*p* < 5 × 10^−8^) were included in the analysis. SNPs significantly correlated with the mode of population covariation were found throughout the genome.

Second, SNPs related to a range of psychiatric and behavioral-cognitive phenotypes were significantly correlated with the mode of covariation (Fig. 1a; FDR-corrected *p* value < 0.05). SNPs correlated with the mode of covariation were distributed across the genome (Fig. 1b). The allele that significantly correlated positively with the genetic canonical score for the top SNP of each psychiatric phenotype encodes an increased risk for that phenotype. In other words, high canonical scores were associated with an increased risk for a wide range of psychiatric phenotypes. These SNPs map to a range of loci (Supplementary Table 3), which in turn map to a range of ontologies (Table 1). In contrast to these associations with psychiatric disorders, SNPs related to neurological disorders showed no such correlations (FDR-corrected *p* value > 0.05) with the notable exception of migraine without aura (*r* = 0.19, FDR-corrected *p* = 0.0015), which parallels the observation that this is the only neurological condition to share heritability with psychiatric disorders [5]. In terms of brain morphology and function, SNPs for facial width and white matter hyperintensities (despite the absence of actual hyperintensities in the PING population) were negatively correlated with the CCA mode. The canonical brain network was found to be virtually identical when SNPs related to brain structure, function, and neurological phenotypes were excluded from the analysis (correlation with the original canonical network *r* = 0.995, *p* < 0.001) demonstrating that these phenotypes do not drive the results. To further explore whether the identified mode of genome–connectome covariation is specific to psychiatric phenotypes, we computed its correlation with SNPs associated with two nonpsychiatric diseases with a well-established genetic component—asthma and type 2 diabetes—and found no significant association (*r* = 0.08 and 0.12, FDR-corrected *p* = 0.84 and 0.066, respectively).

**Table 1:** Results from gene ontology analysis of mapped genes.

	Homo sapiens reference	Fold enrichment	*p* value [Bonferroni-corrected]
Biological processes			
Neuromuscular synaptic transmission	21	>100	6.38 × 10^−11^ [9.28 × 10^−8^]
Regulation of membrane potential	139	32.84	4.29 × 10^−7^ [6.24 × 10^−4^]
Response to drug	81	56.35	3.24 × 10^−8^ [4.71 × 10^−5^]
Response to toxic substance	91	50.16	3.24 × 10^−8^ [8.21 × 10^−5^]
Cellular components			
Ion-channel complex	110	41.49	1.40 × 10^−7^ [5.39 × 10^−5^]
Neuron projection	211	21.63	3.17 × 10^−6^ [1.22 × 10^−3^]
Plasma membrane protein complex	216	21.13	3.54 × 10^−6^ [1.37 × 10^−3^]
Plasma membrane region	424	10.76	8.59 × 10^−5^ [3.32 × 10^−2^]
Receptor complex	177	25.79	1.37 × 10^−6^ [5.27 × 10^−4^]
Molecular function			
Acetylcholine binding/receptor activity	28	>100	2.29 × 10^−10^ [1.05 × 10^−7^]
Extracellular ligand-gated ion-channel activity	85	53.70	4.07 × 10^−8^ [1.87 × 10^−5^]

**Fig. 2:**
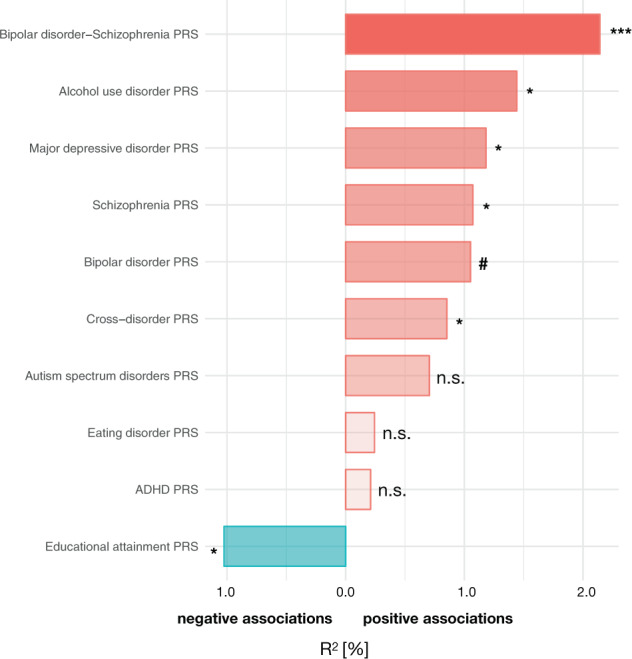
Association (positive toward the right and negative toward the left) between polygenic risk scores (PRS) for psychiatric phenotypes and educational attainment, and the connectomic canonical score. All *p* values are corrected for multiple comparisons: ^#^*p* < 0.1, *p < 0.05, **p < 0.01, ***p < 0.005.

At the polygenic level (Fig. 2 and Supplementary Fig. 3 and Supplementary Table 4), the connectomic canonical score was found to be positively and significantly associated with a PRS for schizophrenia (*R*^2^ = 1.1%, uncorrected *p* = 0.0042, permutation-corrected *p* = 0.030), major depressive disorder (*R*^2^ = 1.2%, uncorrected *p* = 0.0026, permutation-corrected *p* = 0.016), alcohol use disorder (*R*^2^ = 1.4%, uncorrected *p* = 0.00088, permutation-corrected *p* = 0.023), a cross-disorder PRS (*R*^2^ = 0.9%, uncorrected *p* = 0.011, permutation-corrected *p* = 0.030), and a combined bipolar-schizophrenia PRS (*R*^2^ = 2.1%, uncorrected *p* = 4.8 × 10^−5^, permutation-corrected *p* = 0.0010). It was also found to be positively associated with a PRS for bipolar disorder (*R*^2^ = 1.1%, uncorrected *p* = 0.0045, permutation-corrected *p* = 0.068) and for autism spectrum disorders (*R*^2^ = 0.7%, uncorrected *p* = 0.020, permutation-corrected *p* = 0.21) but these associations were not statistically significant after correction for multiple *p*-thresholds. It was not found to be significantly associated with attention-deficit hyperactivity disorder (*R*^2^ = 0.2%, uncorrected *p* = 0.27, permutation-corrected *p* = 0.91) or eating disorders (*R*^2^ = 0.2%, uncorrected *p* = 0.17, permutation-corrected *p* = 0.79). These results strongly suggest that the mode of genome–connectome covariation is driven by SNPs associated with psychiatric phenotypes, and that high canonical scores relate to an increased genetic vulnerability to psychiatric illness.

**Fig. 3:**
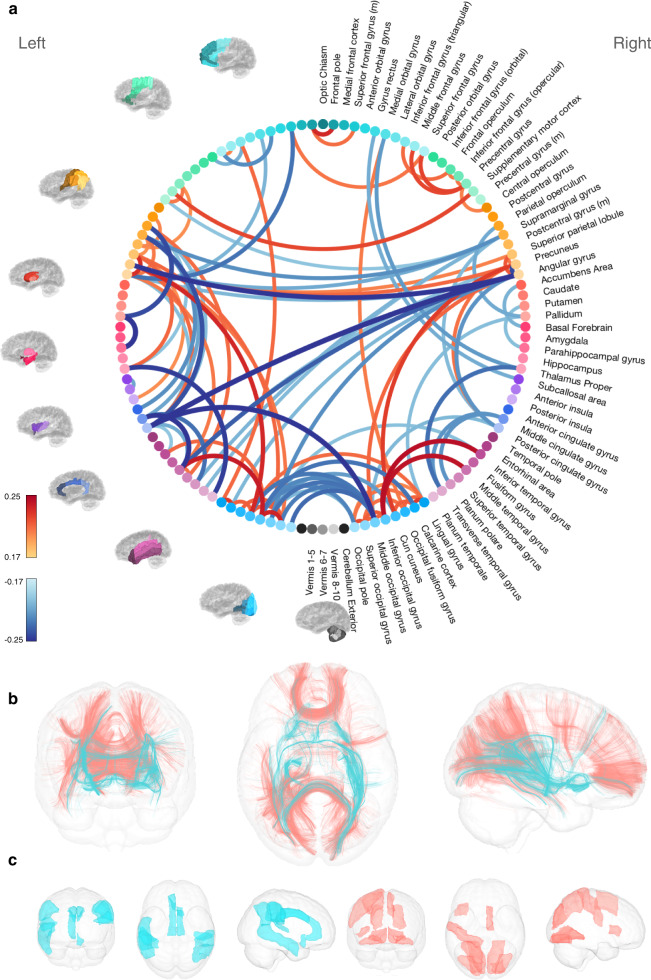
Network of vulnerability to psychiatric illness. **a** Connections with statistically significant canonical strengths form a canonical brain network. Positive (red) and negative (blue) strengths indicate connections whose value respectively increases and decreases with increasing canonical scores. **b** Positive (red) and negative (blue) connections represented in physical space. **c** The hubs in the network show overlap with the default-mode network, a network of cognitive control, and the occipital lobes. In blue and red are hubs of hypoconnectivity and hyperconnectivity respectively (i.e., regions whose average connectivity is respectively lower and higher in individuals with higher canonical scores).

Third, the brain network identified by CCA was made of both positive and negative canonical connection strengths (Fig. 3a, b). By definition, positive (respectively negative) canonical connections tend to be higher (respectively lower) in individuals with high canonical scores. Overall, high canonical scores were negatively correlated with average connection strength (*r* = −0.17, 95% C.I. [−0.24, −0.09], permutation test *p* < 10^−4^) implying that individuals with high canonical scores have on average weaker connectivity. Hubs in the network overlap with three different systems (Fig. 3c; Supplementary Tables 5, 6): the default-mode network (including the angular gyri, posterior cingulate cortices, precuneus, and supramarginal gyri), a network of cognitive control [9, 10] (including the anterior and mid-cingulate cortices, the inferior frontal gyri, and the supplementary motor area), and the occipital cortex. Further details on how the anatomy of the brain vulnerability network overlaps with known systems are provided in the Supplementary Information and Supplementary Fig. 4.

Fourth, the canonical brain network was broadly reproduced when the input SNPs were restricted to specific subgroups of psychiatric phenotypes related to psychosis and autism (correlation with the original canonical network *r* = 0.80, *p* < 0.001), mood disorders (*r* = 0.77, *p* < 0.001), and addiction (*r*=0.54, *p* < 0.001). This finding supports the transdiagnostic nature of the canonical brain network by rejecting the explanation that it is a juxtaposition of subnetworks related to specific categories of psychiatric disorders.

**Fig. 4:**
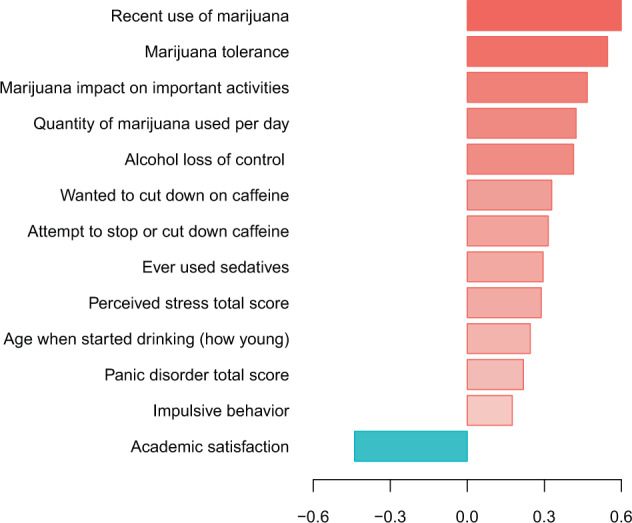
Significant correlations between measurements of social and emotional function and substance exposure and the mode of population covariation. The positive correlations with various behaviors indicate that individuals with higher canonical scores tend to exhibit behaviors related to psychiatric vulnerability.

Fifth, connectomic canonical scores were significantly correlated with measurements of social and emotional function and substance exposure, despite these variables being independent from the inputs to CCA (Fig. 4 and Supplementary Fig. 5; permutation test: *p* = 0.029). Canonical scores were significantly associated with increased marijuana, alcohol, sedatives, and caffeine exposure and misuse, panic disorder score, perceived stress, and impulsive behavior while being negatively associated with academic satisfaction. Mediation analysis revealed that the canonical brain network mediates the influence of genes on these variables (permutation test: *p* = 0.006). This finding implies that even though the PING cohort is a healthy population, individuals with higher canonical scores exhibit behaviors that are related to psychiatric vulnerability.

We also conducted various analyses to test the robustness of the results. The mode of population covariation was found to be robust when reproduced in two mutually exclusive subgroups of participants randomly sampled from the population (correlation between the original and reproduced canonical genetic strengths: 0.89 and 0.93; 95% C.I. [0.87, 0.91] and [0.92, 0.95], respectively; *p* < 0.001 for both; and correlation between the original and reproduced canonical connection strengths: 0.65 and 0.68; 95% C.I. [0.62, 0.68] and [0.65, 0.71], respectively; *p* < 0.001 for both). It was also found to be robust when both genetic and connectomic data (rather than connectomic data alone) were adjusted for admixture genetic variables (correlation between canonical genetic strengths: 0.86; 95% C.I. [0.83, 0.89]; and between canonical connection strengths: 0.95; 95% C.I. [0.95, 0.96]; *p* < 0.001 for both) and when the dataset was restricted to a more homogenous population of European genetic ancestry (correlation between canonical genetic strengths; 0.78; 95% C.I. [0.74, 0.82]; and between canonical connection strengths: 0.74; 95% C.I. [0.71, 0.76]; *p* < 0.001 for both). Furthermore, it was found to be robust when participants exhibiting behaviors related to psychiatric vulnerability were excluded from the analysis (correlation between the original and subject-reduced canonical genetic strengths: 0.92, *p* < 0.001 and between the original and subject-reduced canonical connection strengths: 0.96, *p* < 0.001) demonstrating that these participants do not drive the results but can rather be considered as extremes on the vulnerability spectrum. A breakdown of the mode of genome–connectome covariation by age, sex, scanner manufacturer, and genetic ancestry is also provided in Supplementary Fig. 6. Finally, it was also robust to the number of principal components (from 50 to 150) used in the preprocessing of the connectomic and genomic data (correlation between canonical genetic strengths: 0.86–0.98; and between canonical connection strengths: 0.85–0.96; *p* < 0.001 for both; Supplementary Fig. 7).

## Discussion

The findings presented here support the existence of a structural brain network encoding genetic vulnerability to psychiatric illness across diagnostic categories. Meta-analyses of case-control studies linking brain functional connectivity to psychiatric disorders had suggested that neuroimaging findings are in part shared across psychiatric diagnoses [9]. But such studies are irrevocably limited by the confounders of illness state, its treatment, and its sequelae. The emergence of common psychopathology factors [4, 12], their heritability [12, 50], and neuroimaging correlates [12, 51, 52] further suggested that a shared neurobiology may explain the clinical overlap between psychiatric diagnoses. However, the etiology of these factors remains unclear as they are only partially heritable [4, 12], and their interpretation in terms of vulnerability to psychiatric illness is merely postulated since prospective cohort studies are lacking.

By contrast, the present findings are solely driven by SNPs that confer an established risk for psychiatric disorders. This enables interpretation of the resulting brain network as a “vulnerability network” and the corresponding connectomic canonical scores as “brain vulnerability scores.” Unlike functional brain networks, this vulnerability network is made of white matter fascicles connecting cortical and subcortical areas, which are thought to be state-independent [53]—a necessary condition to qualify as an endophenotype [54, 55]. Whether it represents a true endophenotype or is rather a biomarker [55] will ultimately require longitudinal studies following cohorts with high brain vulnerability scores. But the fact that brain vulnerability scores mediate the impact of genetic vulnerability onto behaviors related to psychiatric illness suggest that the vulnerability network forms part of its pathogenesis [54]. The mechanism by which this shared endophenotype turns into different clinical presentations remains to be discovered. Clinical presentations likely emerge from complex pathophysiological pathways involving interactions between environmental and genetic risks. In such intricate pathways, the vulnerability network might be a central element funneling genetic risks through a common neurobiological root.

The vulnerability network overlaps with a network of cognitive control identified by a meta-analysis of case-control studies across major psychiatric diagnoses [9] and with the occipital cortex. The latter is consistent with the growing evidence for the involvement of occipital cortices in psychopathology as part of a speculated disruption in the selection and processing of sensory information [51]. These overlaps thereby replicate previous findings whilst helping elucidate their etiology. Conversely, previous transdiagnostic findings which diverge from the vulnerability network may result from nongenetic causes such as environmental factors [55]. For instance, the finding of a relation between decreased fronto-occipital white matter connectivity and a common psychopathology factor [12] may reflect its known association with adverse childhood experience [56–58]. Finally, the vulnerability network presents two important specificities, which had not yet been clearly identified as phenotypes shared between psychiatric diagnoses. First, the association between the mode of covariation and a decreased average connectivity suggests that hypoconnectivity is a shared phenotype, which is, at least to a degree, genetically driven and which confers an increased vulnerability to psychiatric illness. This may explain why meta-analyses of case-control studies of white matter integrity more consistently find hypoconnectivity than hyperconnectivity in schizophrenia [59], depression [60], and bipolar disorder [61]. Second, the overlap with the default-mode network may explain why the latter is frequently found to be altered in psychiatric disorders. This overlap supports the suggestion that the default-mode network may also represent a transdiagnostic phenotype [62] while proposing that its disruption may be present before the onset of symptoms and be attributable in part to genetic factors.

The evolution of the brain vulnerability network through the lifespan and its changes in disease remain to be elucidated. Complex gene–environment interactions may substantially alter the association between the vulnerability network and the genetic architecture of psychiatric disorders in adults (indeed this was a major motivation for conducting the present study in a pediatric population). For instance, it might be that the cumulative effect of environmental risk factors for psychiatric illnesses also results in changes to the brain vulnerability network. In that case, high brain vulnerability scores in adults might occur in the absence of genetic predispositions. Conversely, a child with high brain vulnerability score developing in a favorable environment might reach adulthood without ever receiving a psychiatric diagnosis, perhaps reflecting an effect of the favorable environment upon their white matter microstructure. In an initial attempt to explore this question, we used data from 20,827 participants of the UK Biobank in whom suitable MRI data were available [63, 64] (see Supplementary Information and Supplementary Table 7 for details on the methods). Three imaging-derived phenotypes in UK Biobank overlap with the brain vulnerability network (Supplementary Fig. 8) and were predicted to be negatively associated with the genetic canonical score (if the mode of genome–connectome covariation remains unchanged in adulthood): the FA of the forceps major (most significantly) and the two acoustic radiations. We found that the FA of the forceps major was significantly and negatively associated with the genetic canonical scores (*r* = −0.02; *p* = 0.0088, Bonferroni-corrected *p* = 0.026). Moreover, it was significantly lower among participants who had a self-reported psychiatric diagnosis compared with other UK Biobank participants (mean standardized FA −0.056 in “patients” vs. 0.012 in “controls;” 95% C.I. of the difference [0.034, 0.11]; Bonferroni-corrected *p* = 0.00042). These results thus replicate the association of lower occipito-occipital connectivity with genetic predisposition to psychiatric illness (and, even more strongly, with actual psychiatric diagnosis). Further analyses of imaging data from well-phenotyped and genotyped population samples will help clarify the temporal stability and/or dynamic nature of the vulnerability network across the lifespan.

In summary, we discovered a pattern of structural brain connectivity in children which robustly encodes a genetic vulnerability to psychiatric illness which crosses diagnostic boundaries. This brain network shows overlap with a network of cognitive control, the default-mode network, and the occipital cortex, and mediates the impact of genetic risk on behaviors related to psychiatric vulnerability. These findings help explain the shared neurobiology underpinning various psychiatric diagnoses and may contribute to understanding the mechanisms underlying the emergence of psychiatric illness and the interventions aimed at preventing it.

## Supplementary information

Supplementary information

## Data Availability

All MRI preprocessing and estimation of structural connectomes were completed using the image analysis software distributed as the Computational Radiology Kit version 1.6.3 (available at http://crl.med.harvard.edu).

## References

[1] Insel T, Cuthbert B, Garvey M, Heinssen R, Pine DS, Quinn K (2010). Research domain criteria (RDoC): toward a new classification framework for research on mental disorders. Am J Psychiatry.

[2] Kessler RC, Chiu WT, Demler O, Merikangas KR, Walters EE (2005). Prevalence, severity, and comorbidity of 12-month DSM-IV disorders in the National Comorbidity Survey Replication. Arch Gen Psychiatry.

[3] Plana-Ripoll O, Pedersen CB, Holtz Y, Benros ME, Dalsgaard S, de Jonge P, et al. Exploring comorbidity within mental disorders among a Danish national population. JAMA Psychiatry. 2019. 10.1001/jamapsychiatry.2018.3658. 10.1001/jamapsychiatry.2018.3658 PMC6439836 30649197

[4] Caspi A, Houts RM, Belsky DW, Goldman-Mellor SJ, Harrington H, Israel S (2014). The p factor: one general psychopathology factor in the structure of psychiatric disorders?. Clin Psychol Sci.

[5] Anttila V, Bulik-Sullivan B, Finucane HK, Walters RK, Bras J, Brainstorm Consortium (2018). Analysis of shared heritability in common disorders of the brain. Science.

[6] Kapur S, Phillips AG, Insel TR (2012). Why has it taken so long for biological psychiatry to develop clinical tests and what to do about it?. Mol Psychiatry.

[7] Cuthbert BN, Insel TR (2013). Toward the future of psychiatric diagnosis: the seven pillars of RDoC. BMC Med.

[8] Buckholtz JW, Meyer-Lindenberg A (2012). Psychopathology and the human connectome: toward a transdiagnostic model of risk for mental illness. Neuron.

[9] McTeague LM, Huemer J, Carreon DM, Jiang Y, Eickhoff SB, Etkin A (2017). Identification of common neural circuit disruptions in cognitive control across psychiatric disorders. Am J Psychiatry.

[10] Goodkind M, Eickhoff SB, Oathes DJ, Jiang Y, Chang A, Jones-Hagata LB (2015). Identification of a common neurobiological substrate for mental illness. JAMA Psychiatry.

[11] Baker JT, Dillon DG, Patrick LM, Roffman JL, Brady RO, Pizzagalli DA (2019). Functional connectomics of affective and psychotic pathology. Proc Natl Acad Sci USA.

[12] Alnæs D, Kaufmann T, Doan NT, Córdova-Palomera A, Wang Y, Bettella F (2018). Association of heritable cognitive ability and psychopathology with white matter properties in children and adolescents. JAMA Psychiatry.

[13] van den Heuvel MP, Sporns O (2019). A cross-disorder connectome landscape of brain dysconnectivity. Nat Rev Neurosci.

[14] Collin G, Scholtens LH, Kahn RS, Hillegers MHJ, van den Heuvel MP (2017). Affected anatomical rich club and structural–functional coupling in young offspring of schizophrenia and bipolar disorder patients. Biol Psychiatry.

[15] Schmidt A, Crossley NA, Harrisberger F, Smieskova R, Lenz C, Riecher-Rössler A (2017). Structural network disorganization in subjects at clinical high risk for psychosis. Schizophr Bull.

[16] Jernigan TL, Brown TT, Hagler DJ, Akshoomoff N, Bartsch H, Newman E (2016). The pediatric imaging, neurocognition, and genetics (PING) data repository. Neuroimage.

[17] Reus LM, Shen X, Gibson J, Wigmore E, Ligthart L, Adams MJ (2017). Association of polygenic risk for major psychiatric illness with subcortical volumes and white matter integrity in UK Biobank. Sci Rep..

[18] Smith SM, Nichols TE, Vidaurre D, Winkler AM, Behrens TEJ, Glasser MF (2015). A positive-negative mode of population covariation links brain connectivity, demographics and behavior. Nat Neurosci.

[19] Noble KG, Houston SM, Brito NH, Bartsch H, Kan E, Kuperman JM (2015). Family income, parental education and brain structure in children and adolescents. Nat Neurosci.

[20] MacArthur J, Bowler E, Cerezo M, Gil L, Hall P, Hastings E (2017). The new NHGRI-EBI Catalog of published genome-wide association studies (GWAS Catalog). Nucleic Acids Res.

[21] Johnson AD, Handsaker RE, Pulit SL, Nizzari MM, O’Donnell CJ, de Bakker PIW (2008). SNAP: a web-based tool for identification and annotation of proxy SNPs using HapMap. Bioinformatics.

[22] Hamilton CM, Strader LC, Pratt JG, Maiese D, Hendershot T, Kwok RK (2011). The PhenX Toolkit: get the most from your measures. Am J Epidemiol.

[23] Taquet M, Scherrer B, Boumal N, Peters JM, Macq B, Warfield SK (2015). Improved fidelity of brain microstructure mapping from single-shell diffusion MRI. Med Image Anal.

[24] Girard G, Whittingstall K, Deriche R, Descoteaux M (2014). Towards quantitative connectivity analysis: reducing tractography biases. Neuroimage.

[25] St-Onge E, Daducci A, Girard G, Descoteaux M (2018). Surface-enhanced tractography (SET). Neuroimage.

[26] Taquet M, Scherrer B, Commowick O, Peters JM, Sahin M, Macq B (2014). A mathematical framework for the registration and analysis of multi-fascicle models for population studies of the brain microstructure. IEEE Trans Med Imaging.

[27] Peters JM, Sahin M, Vogel-Farley VK, Jeste SS, Nelson CA, Gregas MC (2012). Loss of white matter microstructural integrity is associated with adverse neurological outcome in tuberous sclerosis complex. Acad Radiol.

[28] Lazar M, Weinstein DM, Tsuruda JS, Hasan KM, Arfanakis K, Meyerand ME (2003). White matter tractography using diffusion tensor deflection. Hum Brain Mapp.

[29] Akhondi-Asl A, Warfield SK (2013). Simultaneous truth and performance level estimation through fusion of probabilistic segmentations. IEEE Trans Med Imaging.

[30] Velasco-Annis C, Akhondi-Asl A, Stamm A, Warfield SK (2018). Reproducibility of Brain MRI segmentation algorithms: empirical comparison of local MAP PSTAPLE, FreeSurfer, and FSL-FIRST. J Neuroimaging.

[31] Fonov V, Evans AC, Botteron K, Almli CR, McKinstry RC, Collins DL (2011). Unbiased average age-appropriate atlases for pediatric studies. Neuroimage.

[32] Hotelling H (1936). Relations between two sets of variates. Biometrika.

[33] Mi H, Huang X, Muruganujan A, Tang H, Mills C, Kang D (2017). PANTHER version 11: expanded annotation data from gene ontology and reactome pathways, and data analysis tool enhancements. Nucleic Acids Res.

[34] Martin AR, Daly MJ, Robinson EB, Hyman SE, Neale BM (2019). Predicting polygenic risk of psychiatric disorders. Biol Psychiatry.

[35] Howard DM, Adams MJ, Clarke T-K, Hafferty JD, Gibson J, Shirali M (2019). Genome-wide meta-analysis of depression identifies 102 independent variants and highlights the importance of the prefrontal brain regions. Nat Neurosci.

[36] Stahl EA, Breen G, Forstner AJ, McQuillin A, Ripke S, Trubetskoy V (2019). Genome-wide association study identifies 30 loci associated with bipolar disorder. Nat Genet.

[37] Ripke S, Neale BM, Corvin A, Walters JTR, Farh K-H, Holmans PA (2014). Biological insights from 108 schizophrenia-associated genetic loci. Nature.

[38] Walters RK, Polimanti R, Johnson EC, McClintick JN, Adams MJ, Adkins AE (2018). Transancestral GWAS of alcohol dependence reveals common genetic underpinnings with psychiatric disorders. Nat Neurosci.

[39] Grove J, Ripke S, Als TD, Mattheisen M, Walters RK, Won H (2019). Identification of common genetic risk variants for autism spectrum disorder. Nat Genet.

[40] Watson HJ, Yilmaz Z, Thornton LM, Hübel C, Coleman JRI, Gaspar HA (2019). Genome-wide association study identifies eight risk loci and implicates metabo-psychiatric origins for anorexia nervosa. Nat Genet.

[41] Demontis D, Walters RK, Martin J, Mattheisen M, Als TD, Agerbo E (2019). Discovery of the first genome-wide significant risk loci for attention deficit/hyperactivity disorder. Nat Genet.

[42] Bipolar Disorder and Schizophrenia Working Group of the Psychiatric Genomics Consortium. (2018). Genomic dissection of bipolar disorder and schizophrenia, including 28 subphenotypes. Cell.

[43] Cross-Disorder Group of the Psychiatric Genomics Consortium. (2013). Identification of risk loci with shared effects on five major psychiatric disorders: a genome-wide analysis. Lancet.

[44] Lee JJ, Wedow R, Okbay A, Kong E, Maghzian O, Zacher M (2018). Gene discovery and polygenic prediction from a genome-wide association study of educational attainment in 1.1 million individuals. Nat Genet.

[45] Choi SW, O’Reilly PF (2019). PRSice-2: Polygenic Risk Score software for biobank-scale data. Gigascience.

[46] Benjamini Y, Hochberg Y (2000). On the adaptive control of the false discovery rate in multiple testing with independent statistics. J Educ Behav Stat.

[47] Dai H, Leeder JS, Cui Y (2014). A modified generalized Fisher method for combining probabilities from dependent tests. Front Genet.

[48] Rosseel Y (2012). lavaan: an R package for structural equation modeling. J Stat Softw.

[49] Baron RM, Kenny DA (1986). The moderator–mediator variable distinction in social psychological research: conceptual, strategic, and statistical considerations. J Pers Soc Psychol.

[50] Neumann A, Pappa I, Lahey BB, Verhulst FC, Medina-Gomez C, Jaddoe VW (2016). Single nucleotide polymorphism heritability of a general psychopathology factor in children. J Am Acad Child Adolesc Psychiatry.

[51] Elliott ML, Romer A, Knodt AR, Hariri AR (2018). A connectome-wide functional signature of transdiagnostic risk for mental illness. Biol Psychiatry.

[52] Romer AL, Knodt AR, Houts R, Brigidi BD, Moffitt TE, Caspi A (2018). Structural alterations within cerebellar circuitry are associated with general liability for common mental disorders. Mol Psychiatry.

[53] Park H-J, Friston K (2013). Structural and functional brain networks: from connections to cognition. Science.

[54] Kendler KS, Neale MC (2010). Endophenotype: a conceptual analysis. Mol Psychiatry.

[55] Walters JTR, Owen MJ (2007). Endophenotypes in psychiatric genetics. Mol Psychiatry.

[56] Benedetti F, Bollettini I, Radaelli D, Poletti S, Locatelli C, Falini A (2014). Adverse childhood experiences influence white matter microstructure in patients with bipolar disorder. Psychol Med.

[57] Rodrigo MJ, León I, Góngora D, Hernández-Cabrera JA, Byrne S, Bobes MA (2016). Inferior fronto-temporo-occipital connectivity: a missing link between maltreated girls and neglectful mothers. Soc Cogn Affect Neurosci.

[58] Hanson JL, Adluru N, Chung MK, Alexander AL, Davidson RJ, Pollak SD (2013). Early neglect is associated with alterations in white matter integrity and cognitive functioning. Child Dev.

[59] Kelly S, Jahanshad N, Zalesky A, Kochunov P, Agartz I, Alloza C (2018). Widespread white matter microstructural differences in schizophrenia across 4322 individuals: results from the ENIGMA Schizophrenia DTI Working Group. Mol Psychiatry.

[60] Liao Y, Huang X, Wu Q, Yang C, Kuang W, Du M (2013). Is depression a disconnection syndrome? Meta-analysis of diffusion tensor imaging studies in patients with MDD. J Psychiatry Neurosci.

[61] Wise T, Radua J, Nortje G, Cleare AJ, Young AH, Arnone D (2016). Voxel-based meta-analytical evidence of structural disconnectivity in major depression and bipolar disorder. Biol Psychiatry.

[62] Gong Q, Hu X, Pettersson-Yeo W, Xu X, Lui S, Crossley N (2017). Network-level dysconnectivity in drug-naïve first-episode psychosis: dissociating transdiagnostic and diagnosis-specific alterations. Neuropsychopharmacology.

[63] Alfaro-Almagro F, Jenkinson M, Bangerter NK, Andersson JLR, Griffanti L, Douaud G (2018). Image processing and Quality Control for the first 10,000 brain imaging datasets from UK Biobank. Neuroimage..

[64] Elliott LT, Sharp K, Alfaro-Almagro F, Shi S, Miller KL, Douaud G (2018). Genome-wide association studies of brain imaging phenotypes in UK Biobank. Nature.

