# Supplementary material for: A structural brain network of genetic vulnerability to psychiatric illness
Source: Mol Psychiatry. 2020 May 6;26(6):2089–100. doi: 10.1038/s41380-020-0723-7 (PMC7644622; doi:10.1038/s41380-020-0723-7)
Supplement: Supplementary file 1 — Supplementary information [file 41380_2020_723_MOESM1_ESM.docx]

**Supplemental information**

**SNPs selection and identification of genetic risk**

SNPs associated with psychiatric, neurological, behavior-cognition, or brain structure/function phenotypes were selected from the NHGRI-EBI GWAS Catalog (1) as follows (see Supplementary Fig. 1 for a flowchart). The GWAS catalog version 1.0.1 (as of March 13, 2018) was downloaded. This catalog is an inventory of GWAS studies listing the phenotype investigated, the SNPs identified, the corresponding p-value for each SNP, the Pubmed index of the paper reporting the GWAS study, and the gene that the SNP mapped to. A list of all available phenotypes to which at least one SNP was found to be significantly associated at p < 5×10^-8^ was extracted from the catalog. Phenotypes associated with psychiatric conditions (e.g. schizophrenia), neurological conditions (e.g. migraine), behavior (e.g. smoking behavior), cognition (e.g. mathematical ability), and brain structure/function (e.g. hippocampal volume) were selected (see list in Supplementary Table 2). Phenotypes representing a response to a specific treatment were excluded as they cannot be interpreted as risk factors for specific psychiatric phenotypes. Phenotypes representing old age-specific conditions (e.g. Alzheimer’s disease) were also excluded. This resulted in a list of 96 phenotypes of interest. Inclusion and exclusion of phenotypes into this list were checked by two authors (MT and JMP).

All SNPs associated with the list of phenotypes of interest and with a p-value at publication (as reported in the GWAS catalog) lower than 5×10^-8^ were recorded. If data for these SNPs were available in the PING dataset, then they were included in this study. Otherwise, proxies to these SNPs based on linkage disequilibrium were used instead. All proxies that had a squared correlation coefficient R^2^ > 0.8 with the original SNP of interest and were available in the PING dataset were included in our study. Identifying these proxies was achieved with SNAP (2). If no proxy meeting the conditions were identified, the original SNP of interest was discarded. The latter exclusion also resulted in the discarding of 25 phenotypes which did not have any SNP left for the analysis. These phenotypes had very few SNPs significantly associated with them (average 1.87 SNPs/phenotype). In total, 283 original SNPs and 1594 proxies were selected for each of the 678 participants. SNPs were encoded as additively coded genotypes (0, 1, or 2).

The exact same procedure described above was also applied to identify SNPs and proxies for phenotypes related to asthma and Type 2 diabetes based on the GWAS catalog. All phenotypes from the GWAS catalog containing the word “asthma” and “diabetes” (with the exception of those referring to Type 1 diabetes) were respectively selected.

Population stratification was estimated using ADMIXTURE (3) which returns admixture fractions for every subject and which are encoded as six continuous genetic ancestry factors (GAFs) in the PING dataset. Each GAF corresponds to each of the six major continental populations. By construction, GAFs sum to one and therefore only 5 variables need to be included as covariates. This was performed as part of the wider PING initiative prior to the present study and was included in the PING dataset.

To assess whether participants with high genetic canonical scores are more or less likely to develop a particular phenotype, we identified for each phenotype the SNP most strongly correlated (in absolute value) to the genetic canonical scores. This was done for all phenotypes with at least one SNP significantly correlated with the genetic canonical score (using FDR-corrected p<0.05 as a threshold). If the SNP was a proxy to one of the original SNPs of interest, then the allelic correspondence was confirmed using the LDPair tool from the NIH LDlink platform (4). For each SNP, the corresponding GWAS study was identified from the SNP catalog and revised so that the risk allele and its relation to the phenotype was clearly identified. This step was required because the phrasing of the original GWAS study meant that some phenotypes were ambiguously defined in the GWAS catalog so that the reported risk allele was actually protective against the phenotype of interest (e.g. in one study “neuroticism measurement” referred to a decrease in neuroticism).

**MRI data acquisition and preprocessing**

MRI were acquired using one of 4 different scanners which are represented by a categorical variable in the covariates. For each participant, an MRI acquisition was performed and included: a T1-weighted 3D MPRAGE (Magnetization Prepared Rapid Acquisition GRE) scan using prospective motion correction (PROMO) (5), a 3D T2-weighted variable flip angle fast spin echo scan also using prospective motion correction (PROMO), and diffusion MRI (dMRI) data with 30 gradient directions at a b-value of 1000 s/mm^2^ (TE=91ms, TR=19ms) and one or two images at b=0 s/mm^2^ (also referred to as b_0_ images) with anteroposterior (AP) and posteroanterior (PA) acquisition respectively. Other dMRI acquisition parameters were: matrix size 128×128, resolution 1.875×1.875×2.5 mm^3^.

The dMRI volumes were corrected for magnetic susceptibility distortion based on the pair of b_0_ images with opposite phase-encoding direction (AP and PA) using FSL TOPUP version 5.0.11 (6). Each dMRI volume was checked for signs of residual artefacts (motion, susceptibility, or hardware induced). Motion-related artefact was the only artefact occasionally detected. The volumes containing motion were removed from the dMRI sequence on a per-individual basis. If multiple dMRI series were acquired for an individual, the series with the least number of removed images was selected for further analysis.

For each individual, the T2-weighted image was resampled to the 1×1×1 mm^3^ T1-weighted image, and an intracranial cavity was segmented using a multispectral method (7). Inter-dMRI volume motion/eddy correction was then performed by affine registration of each dMRI volume to the average b_0_ image. The dMRI volumes were finally aligned and up-sampled to the T2-weighted resampled MRI using affine registration and sinc interpolation, and the brain mask obtained by intracranial cavity segmentation was applied to each aligned dMRI volume.

Parcellation of the grey matter into 120 regions was achieved using probabilistic STAPLE (8) applied simultaneously to the T1-weighted and T2-weighted MRI, which was shown to yield accurate and more reproducible results than other standard parcellation methods (9). The same parcellation was also applied to the MNI atlas (10) for visualization purposes.

The multi-tensor model estimated from the dMRI data is composed of up to three anisotropic tensors in each voxel (representing the crossing white matter fascicles) and one isotropic tensor (representing the partial volume of cerebrospinal fluid) whose diffusivity was set to D_0_=3×10^-3^mm^2^/s (the diffusivity of free water at 37°C). An optimization method incorporating a population-informed prior was used to achieve improved estimation accuracy with single non-zero b-value data (11). The population-informed prior was constructed based on dMRI acquired at multiple b-values at the Boston Children’s Hospital and under a protocol approved by the IRB of the latter, in a healthy population of 100 children imaged in an effort to build a normative dataset with a similar age range as the PING cohort (mean age: 11.2 years, s.d. age: 3.83 years).

**Transdiagnostic evaluation and robustness analyses**

To test the hypothesis that the identified brain network is common to the main psychiatric phenotypes (rather than being made of subnetworks each related to a specific psychiatric phenotype), 3 mutually exclusive clusters of psychiatric phenotypes were created (see classification of phenotypes in Supplementary Table 2): mood disorders (9 phenotypes), addiction (19 phenotypes), and psychosis/autism (8 phenotypes). Inclusion and exclusion into these clusters was verified by two authors (MT and PJH). The whole analysis was repeated using as input only the SNPs corresponding to a specific cluster. The network identified using each cluster was compared to the original network by computing the correlation coefficient between their CCA connection strengths.

To test the robustness of the mode of genome-connectome covariation, the PING participants were randomly separated into two subgroups and CCA was conducted in the two subgroups independently keeping 50 principal components to avoid overfitting. The decision to use only half the number of principal components for the replication analysis compared to the primary analysis was motivated by the fact that half the number of observations are used in each replication and hence CCA has an increased likelihood to overfit the data (as discussed in the main text). Furthermore, the robustness of the findings with respect to the number of principal components (Supplementary Fig. 7)—and in particular when this number is set to 50 in the primary analysis—means that the information necessary to discover the mode of genome-connectome covariation is contained in the first 50 principal components of the data matrices. For the first mode of population covariation identified in each subgroup, two Pearson correlation coefficients were calculated: the correlation between the canonical genetic strengths of the subgroup and the corresponding original values and the correlation between the canonical connection strengths in the subgroup and the corresponding original values. Results were deemed robust only if (i) the correlation between the canonical connection strengths of the vulnerability network were positive for both subgroups, (ii) the correlation between the canonical genetic strengths of significant SNPs were positive for both subgroups, and (iii) all four correlation coefficients were statistically significant at the p<0.001 threshold. For the latter, the test was achieved using Matlab corrcoef function.

To test the robustness of the mode of genome-connectome covariation with respect to model specification, we performed the same analysis after correcting both the SNPs and the connection strengths for the genetic ancestry factors (rather than correcting the connection strengths only). As for the replication analysis, two correlation coefficients were calculated: the correlation between the canonical genetic strengths resulting from this new analysis and the corresponding original values and the correlation between the canonical connection strengths resulting from this new analysis and the corresponding original values. The same criteria as mentioned above were applied for the findings to be deemed robust to this change in model specification.

To test the robustness of the mode of genome-connectome covariation with respect to the number of principal components used in the preprocessing of the genomic and connectomic data, we performed the same analysis while changing the number of components from 50 to 150 by steps of 25. The same method as above was used for the findings to be deemed robust to this change of preprocessing. The main manuscript reports the lowest and highest correlations found in the range 50-150 (excluding 100 which naturally leads to a correlation of 1) while all correlations are reported in Supplementary Fig. 7.

To further rule out the possibility that the findings are driven in part by genetic ancestry factors (owing to non-linear effects), CCA was replicated after selecting individuals who have a predominantly European ancestry, i.e. those whose genetic ancestry factor (GAF) related to the European ancestry was larger than all other genetic ancestry factors. As for the other two robustness analyses, the two correlation coefficients (for canonical connection strengths and canonical genetic strengths) were calculated and the finding was deemed to be robust if the above-mentioned criteria were met.

Finally, after showing that the CCA mode of covariation was significantly associated with behavioral variables related to psychiatric vulnerability, an additional robustness analysis was required to assess whether the findings were driven by individuals strongly exhibiting such behaviors. If it were the case, then the identified brain network might be prodromal to psychiatric illness rather than representing a range of vulnerability. To test this hypothesis, we conducted the main analysis again after excluding participants exhibiting any of the behaviors significantly correlated with the CCA mode (as listed in Fig. 4). Participants were excluded if they answered “Yes” to a dichotomous question (e.g. “Did you ever need larger amounts of marijuana to get an effect, or did you ever find that you could no longer get high on the amount you used to use?”) or were among the highest 10% for any continuous scale (e.g. SCARED Panic Disorder Index Score). We also excluded the bottom 10% of individuals in terms of academic satisfaction as this was shown to be significantly negatively correlated with the CCA mode. Robustness was assessed as for the above subsample analysis.

**Data-driven selection of covariates**

Adjustment of connection strengths was based on the following multivariate linear regression:

$$C=C_{0}+\beta_{age} age + \beta_{sex} sex + \beta_{scanner}scanner+\beta_{GAF}GAF.$$

Since all independent variables are defined at the subject level, two types of adjustment can be considered: a subject-level adjustment wherein C represents the average connection strength for a specific subject, and a connection-level adjustment wherein C represents the strength of a specific connection for a specific subject. We therefore proceeded in two steps. First, a subject-level adjustment was performed. The coefficients of the corresponding regression were used to adjust each connection in each subject. Second, a connection-level adjustment was estimated for each connection and, for each independent variable, the regression model was compared to a null regression model in which that independent variable was removed (while the others were kept). Comparison between the full model and the null model was achieved by comparing their Bayesian information criterion (BIC) using the BIC function from R version 3.4.3. The covariate was included in the connection-level covariates if the BIC for the corresponding full model was lower than that of the null model. The final adjustment was achieved by successively applying the subject-level adjustment and a connection-level adjustment in which only those variables leading to a lower BIC were included. This data-driven approach guarantees that independent variables were included in the connection-level adjustment if the data supported such inclusion.

**Association between the brain vulnerability network and social, emotional, and behavioral variables**

The reason for using one-tailed tests for this part of the analysis is that individual associations between the brain vulnerability score and the behavioral variables were collectively analyzed using a combined probability test. This combined approach was favored over individual tests for two reasons:

1. Some of the behavioral variables are highly correlated with one another. An obvious example is tolerance to marijuana which has a correlation of 0.85 with impact of marijuana on important activities. Considering them as independent tests would be misleading.
2. The sample size of the behavioral data is lower than the sample size for the whole population. This is in part because some the PhnX questions do not apply to younger participants. Power calculation shows that only true correlations equal to or above 0.3 can be detected with a statistical power of 90%, a p-value threshold of 0.05 and a sample size of 117. Combining behavioural variables in a single test aims at increasing statistical power.

Given the motivation to perform a combined probability test, the *input* p-values to this test had to be obtained from one-tailed tests. Otherwise, the combined probability test might return a significant but meaningless result. For instance, if the vulnerability network was significantly *positively* correlated with marijuana consumption (e.g. with p=0.02) and significantly *negatively* correlated with perceived stress (e.g. with p=0.03), then the result of the combined probability test might be significant yet meaningless in terms of its interpretation as “behavioral vulnerability”. On the other hand, if one-tailed tests are used with the relevant tail selected to represent behavioral vulnerability, then the negative correlation with perceived stress would have a p-value of 0.985 (=1-0.03/2) and the combined probability test would not yield a significant result. Using one-tailed t-tests as *inputs* to the combined probability test guarantees that a significant result can only be achieved if there is a significant association with behaviors related to psychiatric vulnerability and not just any (mixed) association between the brain vulnerability network and behavioral variables.

**Identifying overlaps between the brain vulnerability network and known brain systems**

The brain vulnerability network has a distributed anatomy which cannot simply be summarized as a superposition of known systems/brain networks. It rather represents a newly identified phenotype which can be used to assess novel associations with higher-order phenotypes (such as clinical features) and which can also be used as a covariate in case-control or cohort studies of specific diagnoses.

However, identifying overlaps between this network and known brain systems serves two purposes. First, it helps partially dissect its anatomy to better understand its association with psychiatric illnesses. Second, it provides potential explanations for the alterations of these brain systems in psychiatric illnesses.

The vulnerability network hubs are cortical and subcortical grey matter regions whose connections with other regions play an important role in driving the brain vulnerability score towards higher values. Focusing on hubs (i.e. brain regions) rather than individual connections enables us to identify overlaps with other brain systems across imaging modalities as it has been used in other studies [12].

We first identified which of the 120 regions which compose the parcellation used in the present study are part of the anatomy of the three brain systems as follows.

*Occipital cortex*: Left Calcarine cortex, Left Occipital fusiform gyrus, Left Cun cuneus, Left Inferior occipital gyrus, Left Middle occipital gyrus, Left Superior occipital gyrus, Left Occipital pole, Left Cerebellum Exterior, Right Superior occipital gyrus, Right Middle occipital gyrus, Right Inferior occipital gyrus, Right Cun cuneus, Right Occipital fusiform gyrus, Right Calcarine cortex, Right Lingual gyrus, and Right Planum temporale.

*Default mode network* (based on a landmark study by Greicius et al. [13]): Posterior cingulate cortex, anterior cingulate cortex, inferior parietal cortex (which in our network corresponds to its constituents: the supramarginal gyrus and angular gyrus), and medial prefrontal cortex

*Network of cognitive control* (based on Table S3 of the original paper by McTeague et al. [14]): dorsal anterior cingulate and anterior mid-cingulate cortex (*anterior cingulate gyrus* and *middle cingulate gyrus)*, pre-supplementary motor area (*supplementary motor cortex*), right insula (*right posterior insular* and *right anterior insula*), right intraparietal sulcus ([*no equivalence in our parcellation*]), left prefrontal cortex extending from mid-dorsolateral prefrontal to premotor cortex (*Left Superior frontal gyrus*).

We then identified where on the distribution of node degrees the different regions of these three brain systems map (Supplementary Fig. 4). These maps show that the nodes within the occipital cortex occupy most of the highest positive degrees (i.e., they are positive hubs in the network). The default mode network and the network of cognitive control mostly map onto the highest negative degrees and absolute degrees.

The statistical significance of these overlaps can be assessed by testing the null hypothesis that their degrees are equal on average to other degrees (because the default mode network and the network of cognitive control both have high negative and absolute degrees, they are each excluded when testing the significance of the other). This was achieved using two-sample t-tests and the results are shown in Supplementary Table 6, which shows that these three brain systems show significant overlaps with the brain vulnerability network.

**List of selected SNPs**

rs1051730, rs1451240, rs2884808, rs1012053, rs10994397, rs4765914, rs12576775, rs2731517, rs1064395, rs4650608, rs6746896, rs13094687, rs1553656, rs4948418, rs420259, rs1944449, rs10501439, rs13166360, rs1906252, rs9375195, rs4624519, rs4234258, rs9821223, rs10275045, rs6954673, rs6978048, rs6954521, rs10233560, rs1565922, rs2941504, rs2941503, rs1810132, rs12150298, rs10267593, rs10278591, rs174576, rs11085829, rs931067, rs2710323, rs1108842, rs2535627, rs20585, rs215001, rs214952, rs127196, rs3782638, rs1860961, rs1154153, rs12587781, rs1263645, rs1551570, rs2834188, rs7075349, rs198806, rs6918586, rs4307059, rs4141463, rs6537825, rs926938, rs11589568, rs6913660, rs6904071, rs6938200, rs7745603, rs6932590, rs12807809, rs9960767, rs2297786, rs4532960, rs4307650, rs7092200, rs12413409, rs12411886, rs11191425, rs4409766, rs3824754, rs17115100, rs4309482, rs1625579, rs10503253, rs1635, rs10489202, rs16887244, rs17749927, rs13194781, rs10484399, rs6904596, rs13197574, rs175597, rs200953, rs149990, rs12666575, rs4604142, rs1986252, rs7527939, rs7096169, rs3800913, rs1782812, rs2660300, rs1702292, rs2159100, rs10774035, rs4298967, rs7893279, rs10828679, rs4129585, rs2340400, rs1885246, rs6424546, rs1338654, rs7111478, rs1991899, rs1118137, rs6590540, rs10414830, rs6909, rs2269873, rs735273, rs2373000, rs2123133, rs1901521, rs7731256, rs1562961, rs10075318, rs1452057, rs11746217, rs2060833, rs1376898, rs7737204, rs1562960, rs6873738, rs10069193, rs1502844, rs7734926, rs9327836, rs709372, rs2071508, rs12991836, rs709937, rs778341, rs884808, rs1058304, rs34635, rs11098403, rs1261117, rs10910078, rs1009080, rs267700, rs11210892, rs1938570, rs4378243, rs12132780, rs4844394, rs2796267, rs11682175, rs10205801, rs17194490, rs12495352, rs4243834, rs2033377, rs1430895, rs9862290, rs7432375, rs1351235, rs215410, rs10520163, rs1106568, rs1501357, rs10454909, rs2161123, rs3797680, rs3849046, rs2789588, rs1572209, rs10241415, rs982256, rs38752, rs214469, rs214467, rs214463, rs214460, rs2529489, rs13230189, rs728054, rs320704, rs12534625, rs11780592, rs6471814, rs867743, rs6471803, rs7463315, rs6471835, rs7845438, rs6471845, rs4440623, rs6990717, rs627542, rs548902, rs13267290, rs7002308, rs4735158, rs7127372, rs11027859, rs11027860, rs11027845, rs9420, rs1439513, rs6589377, rs7927176, rs10791098, rs3802924, rs2007044, rs302321, rs302342, rs1727302, rs4460848, rs1790098, rs1106240, rs2693698, rs2296482, rs2414716, rs12595508, rs2414718, rs11854700, rs1971791, rs8042374, rs950169, rs4702, rs9933832, rs7201930, rs9938117, rs8058295, rs1861192, rs6497520, rs7185124, rs7193701, rs1975802, rs2418736, rs7205935, rs6499157, rs216219, rs11655813, rs2281727, rs9945732, rs919803, rs3786800, rs1004046, rs6065094, rs9656169, rs4730430, rs7783665, rs1396176, rs6940230, rs696520, rs9810089, rs11717954, rs4521165, rs9836231, rs6802702, rs10935183, rs1789589, rs1792707, rs1792708, rs10305724, rs11993663, rs3771206, rs2196806, rs3771211, rs7573382, rs2194545, rs978212, rs876701, rs12574668, rs12283172, rs2291487, rs7951579, rs12574250, rs7112229, rs3802888, rs2902858, rs11819869, rs7125907, rs7484002, rs7109698, rs2171668, rs7130141, rs7122039, rs2153960, rs3800229, rs1935949, rs4946936, rs2764264, rs9400239, rs3752591, rs9311474, rs352163, rs11127125, rs4666014, rs4666022, rs7595986, rs10175508, rs3792253, rs10491964, rs11638290, rs12899981, rs2271431, rs11635597, rs3748376, rs12635178, rs13107325, rs13198474, rs6915678, rs1028308, rs9295730, rs6921388, rs13211507, rs133047, rs9939422, rs4353494, rs7203355, rs210152, rs11683083, rs281783, rs769950, rs12613687, rs3001723, rs301798, rs301789, rs2708633, rs6678140, rs10779702, rs3798149, rs3788568, rs4522708, rs217309, rs217323, rs217291, rs217290, rs1979, rs3857546, rs13195509, rs16891334, rs13212534, rs1536501, rs1536500, rs942496, rs3957165, rs652049, rs2296569, rs9393777, rs13219354, rs1046778, rs848291, rs10170257, rs10883766, rs17114803, rs4146429, rs2031604, rs4148865, rs6740981, rs4673634, rs1106400, rs10445792, rs1727309, rs655293, rs883263, rs7304782, rs999494, rs6802636, rs730243, rs938682, rs12910984, rs6495309, rs9292918, rs9607658, rs10108725, rs4583255, rs11901, rs6430491, rs6500596, rs6994019, rs10955542, rs2767713, rs499472, rs12564425, rs11210235, rs4113050, rs11210266, rs12742409, rs11210187, rs6692884, rs7522520, rs10185472, rs4666020, rs154073, rs256002, rs2043273, rs2268439, rs11959184, rs951475, rs4856666, rs7615033, rs758129, rs322005, rs321999, rs12665877, rs10148671, rs13266463, rs314280, rs314262, rs4946651, rs10940346, rs783540, rs223313, rs4698874, rs223447, rs150895, rs223420, rs223482, rs223344, rs223346, rs2247870, rs1421750, rs193495, rs13254942, rs6999466, rs4799088, rs4790084, rs7214541, rs2131431, rs4958573, rs4463219, rs4310018, rs17565365, rs4464293, rs7570682, rs4793885, rs1566522, rs12511877, rs17627811, rs7597593, rs10266871, rs17571979, rs974074, rs1042992, rs714975, rs6801189, rs4855015, rs1806190, rs13096176, rs7647398, rs1805610, rs1805604, rs1805589, rs1805563, rs1805562, rs1835720, rs2312147, rs2717001, rs10188070, rs1460255, rs1402398, rs12991325, rs12620940, rs1402399, rs2953441, rs2717048, rs10496076, rs989613, rs6975286, rs12629572, rs739431, rs2282751, rs1547643, rs816278, rs10774610, rs10234844, rs2222544, rs13221252, rs2866277, rs6961345, rs10499812, rs6975279, rs10268924, rs10256186, rs4643, rs6671606, rs1049296, rs8177313, rs1534166, rs3811647, rs1229984, rs12042938, rs1545843, rs12054895, rs7647854, rs2715147, rs2888019, rs2522833, rs4074418, rs4074415, rs9883177, rs3744017, rs1060105, rs4759409, rs8756, rs7968902, rs6581612, rs4273712, rs16862377, rs2324337, rs7333056, rs9548805, rs9315703, rs9548830, rs7164409, rs9525638, rs2254595, rs2169520, rs16997087, rs17819300, rs4747011, rs2224003, rs17024684, rs9834692, rs9883474, rs3771863, rs10485022, rs2618516, rs7590720, rs1789891, rs4478858, rs572971, rs1768127, rs1707972, rs1768254, rs6773938, rs1708059, rs12490768, rs1606473, rs4366594, rs4337169, rs7946010, rs621246, rs615358, rs214922, rs752028, rs2251219, rs8034191, rs13273442, rs6474414, rs10958725, rs4933206, rs3736329, rs2305797, rs6265, rs3025316, rs8102683, rs1861046, rs2192362, rs7950811, rs4315640, rs1572299, rs11790829, rs12201676, rs2799573, rs70018, rs214976, rs7004633, rs133885, rs11743006, rs349050, rs10490092, rs10490093, rs1950160, rs1380703, rs1441640, rs10883855, rs4233964, rs11682091, rs525798, rs1533208, rs1512660, rs2055966, rs1512514, rs13259216, rs1039916, rs2428, rs1510934, rs2409096, rs12376216, rs10959797, rs10809476, rs2171661, rs10491952, rs10809523, rs7047280, rs4977611, rs10757412, rs1187256, rs6799284, rs2409745, rs2409722, rs2409718, rs9657519, rs2409691, rs5758264, rs3818003, rs4820425, rs926914, rs13053242, rs10960067, rs10809527, rs4360891, rs9672504, rs3935685, rs12938775, rs1786971, rs9964724, rs12956949, rs7505519, rs1411731, rs1544161, rs2736372, rs958648, rs3808513, rs2164273, rs9286062, rs6601578, rs6601573, rs1520636, rs4938021, rs7837587, rs12546366, rs6984496, rs10096421, rs11783247, rs4240673, rs2945230, rs2948300, rs2898290, rs2409798, rs13280813, rs10283145, rs17765901, rs435581, rs1073913, rs6601522, rs6993841, rs10503426, rs2898295, rs12678938, rs555200, rs7833387, rs4799461, rs7942486, rs2157977, rs10254445, rs11769491, rs2299517, rs2299519, rs17683391, rs6951643, rs4731330, rs1557646, rs1542211, rs7696796, rs9287411, rs1895694, rs1377454, rs2042555, rs3820716, rs1234413, rs10928374, rs10512249, rs7809518, rs4969391, rs929579, rs2193596, rs7132057, rs10850135, rs9858071, rs895941, rs9867227, rs2051293, rs7545247, rs12123067, rs12377084, rs10429595, rs10756286, rs9298995, rs499188, rs708228, rs12790660, rs10511261, rs4476839, rs6922758, rs9387954, rs17058157, rs12445568, rs7993004, rs7235757, rs3741475, rs727164, rs7776980, rs10276758, rs7782815, rs11240962, rs6865111, rs6882046, rs7299040, rs4766899, rs11068917, rs9943819, rs16948234, rs2280711, rs4772087, rs199933, rs1115012, rs2383772, rs1521736, rs1521739, rs1996774, rs10812849, rs240764, rs802467, rs724224, rs11505922, rs10757761, rs7024629, rs7039397, rs10812864, rs6476091, rs10968740, rs976697, rs10812854, rs1400861, rs932184, rs7569110, rs10932551, rs1400863, rs7603357, rs17711053, rs16852238, rs297367, rs7941820, rs10790862, rs12314392, rs1450832, rs1919159, rs1818991, rs707124, rs7567451, rs7202054, rs6060043, rs6060034, rs2378249, rs2378199, rs7110863, rs7937151, rs7108081, rs7938812, rs7105462, rs7242858, rs8083506, rs983294, rs11876458, rs6479494, rs1563245, rs2152697, rs4585149, rs848293, rs4587942, rs8179610, rs11124367, rs10181985, rs9647718, rs6002626, rs2284087, rs7245, rs133335, rs6519301, rs215596, rs929456, rs10236197, rs215622, rs215605, rs412876, rs215632, rs739496, rs2301621, rs6490162, rs630512, rs616668, rs10931897, rs3769476, rs295140, rs10511083, rs6804845, rs2117153, rs2326319, rs2875907, rs8017172, rs1953352, rs8021018, rs6060599, rs6060627, rs6089075, rs879982, rs666845, rs505998, rs494071, rs601826, rs641838, rs511740, rs581178, rs2436152, rs1432053, rs6573040, rs11665242, rs10502966, rs2513466, rs12894779, rs3856228, rs10922924, rs2271933, rs4949455, rs1474182, rs2292988, rs4949457, rs2364535, rs12032756, rs12024263, rs1887402, rs501299, rs11210869, rs6674176, rs12124523, rs2568955, rs12143898, rs6678734, rs1931262, rs11583841, rs17372140, rs10494040, rs4970760, rs4970729, rs12073497, rs1144593, rs11588857, rs12739630, rs12733930, rs11580728, rs10926978, rs12741781, rs10926958, rs2163048, rs11686372, rs4303732, rs13010010, rs10175405, rs10180461, rs13405986, rs13422673, rs10932132, rs12478029, rs7309, rs4500960, rs4664442, rs2909448, rs4637136, rs7592562, rs7566330, rs1455335, rs748832, rs6801153, rs13324142, rs2276852, rs13063312, rs12107418, rs12107252, rs9834639, rs2633958, rs3197999, rs13085791, rs9858280, rs4625, rs11706370, rs9837520, rs17304079, rs11720121, rs6549400, rs9809213, rs7374494, rs2884484, rs6818637, rs2189234, rs2454206, rs2726491, rs7664704, rs11724252, rs34320, rs187270, rs34318, rs382784, rs6569077, rs2039722, rs10484867, rs10484868, rs6911407, rs2802288, rs7792396, rs6975134, rs2042000, rs3757966, rs1063739, rs2721195, rs10108150, rs4925811, rs10965819, rs2781530, rs1831555, rs1977552, rs11138947, rs7871404, rs10118793, rs10761035, rs10761013, rs10818605, rs10760193, rs10760198, rs4564007, rs4363310, rs9411334, rs7026534, rs7923609, rs4379723, rs10509186, rs1541046, rs7080055, rs4244350, rs812799, rs1484237, rs1332718, rs10884126, rs12248627, rs10741363, rs4615986, rs10830562, rs10741362, rs1894137, rs1784135, rs568668, rs3748256, rs693364, rs10765777, rs3808977, rs7110786, rs10772783, rs747527, rs10845987, rs1701704, rs773107, rs9634624, rs2476736, rs2478288, rs2251829, rs11157930, rs941721, rs2295682, rs3794452, rs9788566, rs974471, rs10129426, rs2289328, rs11071848, rs12917564, rs7180542, rs3743171, rs870022, rs2727102, rs2572209, rs9635366, rs3784448, rs892650, rs8028238, rs7171289, rs2289045, rs1501372, rs1501371, rs4788102, rs8049439, rs4788084, rs8088313, rs6505776, rs7234960, rs570505, rs578208, rs6505780, rs527839, rs474337, rs1786263, rs1893662, rs3794923, rs132582, rs9616812, rs9628185, rs1347852, rs11662175, rs7973260, rs6012564, rs4810909, rs3091529, rs2075678, rs6066968, rs4810898, rs10809521, rs782207, rs782239, rs782236, rs2422221, rs4935126, rs3764002, rs17106852, rs2078371, rs10166942, rs6431648, rs10490012, rs9349379, rs9398148, rs6899647, rs12207471, rs3860243, rs9486181, rs6478241, rs2160875, rs11172113, rs4705901, rs3776016, rs4705890, rs3756287, rs601441, rs477086, rs12513768, rs31239, rs11242069, rs77938, rs7706785, rs924434, rs1556876, rs1115535, rs4583879, rs13170178, rs7722574, rs4629584, rs9427232, rs6668066, rs1127091, rs1139620, rs4341393, rs9426935, rs4596938, rs11264680, rs11264532, rs11264743, rs11583896, rs4851269, rs6740838, rs1160542, rs2777888, rs7731099, rs7711446, rs12515820, rs12515179, rs7711528, rs6866995, rs3935213, rs4958191, rs6557171, rs6141319, rs242997, rs10908474, rs2415984, rs13159331, rs10055714, rs4975013, rs1260326, rs1375547, rs9822731, rs2196096, rs9309982, rs9819476, rs1821349, rs9811546, rs698, rs1789924, rs2851300, rs941752, rs10133305, rs8009527, rs1363605, rs2173201, rs1442492, rs1614972, rs10799590, rs6044001, rs3758171, rs3936340, rs7805864, rs958404, rs889826, rs6955240, rs17695373, rs1472966, rs2781540, rs1761287, rs10518440, rs9464203, rs13200042, rs12530462, rs4839837, rs12208449, rs926276, rs2472890, rs2472888, rs6922111, rs17312661, rs213230, rs6124071, rs1499895, rs6779258, rs11720523, rs9813516, rs11223648, rs16966381, rs1833161, rs2077923, rs4932370, rs2226669, rs6442165, rs12522297, rs6923139, rs3617, rs489770, rs534987, rs17552495, rs6882166, rs36341, rs427691, rs4714675, rs7774567, rs11587000, rs13317, rs7821392, rs2306899, rs1945885, rs12361324, rs221903, rs3814871, rs917065, rs353547, rs2581806, rs2244461, rs2581777, rs1204077, rs3801999, rs2299304, rs10750025, rs13218591, rs1011665, rs13220817, rs1883403, rs1080500, rs12176317, rs9379859, rs9379858, rs16891725, rs7746199, rs1475753, rs7578761, rs12987780, rs12898656, rs12910951, rs12909292, rs2163492, rs2033404, rs1440606, rs777707, rs777709, rs11779061, rs7011229, rs11082972, rs6508210, rs7562587, rs4438512, rs958217, rs1580022, rs2392056, rs2134046, rs1538482, rs1569750, rs1983639, rs6095360, rs12072199, rs17412284, rs11588952, rs4949448, rs2271928, rs2176807, rs10889959, rs4660192, rs12408956, rs2428963, rs17474535, rs3828150, rs2842186, rs12410155, rs7520053, rs3791046, rs12354267, rs673253, rs2989868, rs4424536, rs12082973, rs854286, rs2420178, rs9988609, rs7414744, rs9436866, rs975480, rs3101336, rs1157072, rs12125471, rs11587434, rs1620977, rs1486096, rs2821296, rs1675356, rs1194269, rs1194265, rs9424977, rs1776012, rs1870676, rs12139692, rs962231, rs479934, rs12120564, rs626398, rs578096, rs1342782, rs1335732, rs6664676, rs1931254, rs2700338, rs2802530, rs6692914, rs11164719, rs3820667, rs4083386, rs10863899, rs4665251, rs2176264, rs9798012, rs2879609, rs925229, rs6543658, rs387780, rs13021125, rs1364524, rs6545625, rs13030292, rs2245273, rs1405819, rs13012916, rs243071, rs1011407, rs733628, rs7565301, rs6748040, rs4641966, rs6720094, rs6546824, rs6718864, rs7557055, rs7557285, rs768851, rs6718690, rs3112256, rs2175792, rs4850948, rs2309920, rs4851313, rs2009094, rs2118280, rs13032879, rs13398213, rs2942904, rs870321, rs2067783, rs764828, rs4851250, rs11692215, rs4851287, rs2309818, rs726000, rs10165214, rs6742579, rs6744254, rs16838536, rs13390547, rs4638741, rs17244660, rs1471362, rs10192369, rs6432667, rs7565794, rs2284872, rs7586673, rs2082136, rs6720187, rs12987781, rs2350807, rs17199249, rs2138380, rs4130782, rs2001209, rs13325754, rs9876561, rs7431278, rs4131864, rs1317140, rs3749237, rs1352889, rs4855882, rs9829155, rs13100903, rs2624839, rs2526388, rs2526748, rs4688758, rs6765484, rs11713193, rs2681781, rs2230590, rs6446298, rs9855505, rs6802890, rs1010554, rs1133415, rs2581795, rs830640, rs2875528, rs10511271, rs13088101, rs1343700, rs4974424, rs2084400, rs13064915, rs6439699, rs3912122, rs11921899, rs10004503, rs1346075, rs730934, rs1383724, rs11131666, rs787361, rs12498631, rs2164300, rs11945232, rs10516930, rs13114738, rs13116385, rs13129951, rs17032400, rs17199964, rs230489, rs10010325, rs2726513, rs2726507, rs2726485, rs2726490, rs2647259, rs2636726, rs2713861, rs2726503, rs2726471, rs1391438, rs1391441, rs7678440, rs2726459, rs3796627, rs3749561, rs4696283, rs6535818, rs12331037, rs1561915, rs1946999, rs6536370, rs4690912, rs6858744, rs7666007, rs9998332, rs4690916, rs1045352, rs1445981, rs7714712, rs6868457, rs12523278, rs12517174, rs1460958, rs4235481, rs6872863, rs1422189, rs7721099, rs16903285, rs2304607, rs12054920, rs4518438, rs17558396, rs4327622, rs7720113, rs157566, rs662339, rs818939, rs749896, rs1596431, rs12189452, rs2301012, rs1008661, rs6894052, rs3797696, rs376433, rs686349, rs402641, rs403133, rs1364931, rs2436391, rs6867870, rs7737543, rs2189659, rs7268, rs830230, rs31772, rs830383, rs2731664, rs538827, rs554671, rs573082, rs588062, rs10807604, rs17770697, rs975303, rs1322537, rs2154219, rs16891315, rs16891235, rs6940007, rs3734523, rs7748167, rs1892250, rs9379897, rs1796518, rs9467632, rs198828, rs198838, rs1572982, rs2032447, rs2093169, rs7756567, rs9358946, rs3799380, rs9379895, rs7740197, rs4320356, rs2237236, rs4712984, rs10946813, rs12199613, rs12190473, rs3736781, rs6456735, rs1407045, rs1796520, rs1624440, rs9361491, rs9443645, rs9490788, rs1386550, rs9401295, rs12216178, rs1376503, rs9388349, rs7754741, rs9489926, rs4839713, rs1933720, rs1906251, rs2450512, rs10872224, rs13194250, rs4412207, rs9372650, rs6940021, rs2505059, rs4557524, rs6924808, rs1268166, rs6568547, rs6927268, rs9285397, rs9480861, rs9388489, rs9372840, rs1490388, rs6979354, rs9648380, rs1050331, rs1065646, rs10252662, rs756912, rs1468163, rs2944822, rs2944814, rs2968538, rs10486883, rs9655780, rs4731367, rs10487490, rs12706812, rs7809567, rs1043595, rs6973256, rs12707093, rs1030268, rs1364503, rs2059376, rs9649045, rs2432641, rs6956399, rs10263196, rs2971971, rs2971953, rs2971955, rs4731990, rs2971970, rs4731970, rs2041996, rs12156096, rs10503484, rs10105802, rs13253386, rs1478026, rs3024239, rs13278931, rs11783562, rs11777025, rs17302392, rs13262793, rs13259522, rs13259607, rs1481045, rs11781016, rs7825562, rs10089969, rs13273012, rs2083844, rs720444, rs2930536, rs12549680, rs11782215, rs4977607, rs10780928, rs895606, rs6559863, rs10992768, rs10821148, rs3813388, rs10821160, rs10156602, rs10761229, rs1055710, rs12379660, rs4743923, rs10761240, rs10761243, rs10821123, rs3005191, rs702980, rs1329255, rs3005182, rs10761768, rs9629895, rs3211105, rs907, rs10733788, rs7098614, rs6479889, rs10465990, rs12768534, rs10822186, rs6479894, rs4746195, rs868798, rs12773283, rs1009984, rs1010889, rs10822129, rs1891275, rs1044258, rs11191161, rs4919626, rs1890060, rs7085239, rs10500759, rs10831905, rs2241857, rs4415740, rs7930077, rs4757956, rs12289262, rs10830568, rs10501713, rs2155076, rs1540170, rs2213084, rs7926034, rs1479119, rs7312042, rs7954210, rs983798, rs7135743, rs2193749, rs7138997, rs1012642, rs10880880, rs10875914, rs17123764, rs10876864, rs7954957, rs6539284, rs12313068, rs937564, rs585522, rs4148863, rs1790094, rs949143, rs10773921, rs7296418, rs12552, rs636690, rs9537782, rs7326551, rs9537784, rs9537888, rs9527754, rs633158, rs7323027, rs337948, rs4981690, rs10450901, rs10138733, rs4981691, rs11849844, rs4981684, rs1957476, rs2333611, rs3783297, rs2273813, rs6571317, rs1959433, rs9322846, rs4261436, rs1051695, rs10047954, rs11625806, rs11624944, rs11627968, rs2001355, rs1275415, rs10148349, rs4904523, rs4904522, rs965770, rs17692512, rs2071407, rs1456295, rs1840707, rs12324531, rs4775954, rs4774594, rs10519317, rs1471072, rs1902590, rs2607117, rs8028518, rs4778672, rs1501367, rs3785229, rs183008, rs350255, rs4781183, rs200536, rs2343606, rs2547033, rs2650492, rs151228, rs12446550, rs6565259, rs8045689, rs2908792, rs9921587, rs13332406, rs8043918, rs8056923, rs7189819, rs9925415, rs12708923, rs9930344, rs1837020, rs806739, rs806742, rs4788449, rs2335712, rs4788585, rs2074103, rs9906189, rs7224296, rs9911578, rs3744279, rs17202270, rs17202291, rs8071332, rs11083241, rs1431197, rs9959914, rs4939723, rs4270245, rs11874659, rs11082974, rs1010360, rs1125627, rs2292043, rs1943097, rs1943093, rs2134297, rs4453579, rs10502976, rs11876154, rs11876782, rs10853631, rs11082993, rs1394466, rs6417121, rs4129439, rs4414555, rs7236339, rs10403210, rs12985909, rs4813165, rs6110407, rs394643, rs3848715, rs2024595, rs6012542, rs6067054, rs6090919, rs6095298, rs6067037, rs2145839, rs2145263, rs6015543, rs6026998, rs1389994, rs714026, rs3788557, rs909674, rs4407350, rs10854884, rs5770820, rs2139539, rs4949459, rs1026997, rs11209951, rs4949969, rs11165468, rs3738773, rs2309796, rs11686880, rs2309798, rs2164847, rs1030902, rs2241810, rs4850931, rs1030901, rs3748930, rs4149518, rs2241809, rs4149510, rs3828193, rs2241811, rs4149517, rs2099611, rs1115910, rs6749757, rs2883059, rs6774354, rs7061, rs1061474, rs1138536, rs2856237, rs2289247, rs3755806, rs11177, rs6762813, rs6976, rs4698932, rs26949, rs9490133, rs12214442, rs4415209, rs1149558, rs3823572, rs7782545, rs12707127, rs6954859, rs6967343, rs7864397, rs2398856, rs2001610, rs11534279, rs2293445, rs10783299, rs11168839, rs10846489, rs7139321, rs6561938, rs2074404, rs415430, rs12953529, rs745086, rs1970546, rs10770107, rs10846216, rs11056567, rs12231793, rs719714, rs739215, rs10494950, rs7868992, rs10901815, rs17624662, rs1823068, rs2148710, rs4280164, rs681524, rs1940475, rs3765620, rs6568686, rs17080774, rs17794346, rs10510007, rs6602398, rs1709393, rs1937600, rs12662873, rs2898883, rs1433375, rs12692189, rs6711222, rs6493265, rs7163647, rs7176566, rs12898994, rs921764, rs715693, rs35289

**Analysis of the brain vulnerability network in UK Biobank**

To gain insight into the association between the genetic predisposition to psychiatric illness and brain microstructural connections in adults, we conducted an additional analysis among 20,827 participants of the UK Biobank. Whole-brain tractography was not available as a phenotype in the current UK Biobank data. But the fractional anisotropy for 27 white matter tracts were available as imaging-derived phenotypes (IDPs). This opens the opportunity to assess the association between the genetic canonical score and white matter connections that overlap with the brain vulnerability network. This analysis was carried out in 5 steps:

1. We calculated the value of these 27 IDPs in the PING population.
2. We assessed within the PING population whether these IDPs were significantly correlated with the genetic canonical scores (and if so whether the correlation was positive or negative). This provides testable predictions about associations between genetic predisposition to psychiatric illnesses and IDPs under the assumption that the mode of genome-connectome covariation remains unchanged in adulthood. Significant associations (after Bonferroni corrections for multiple comparisons across the 27 IDPs tested) are due to significant overlap between the IDPs and the brain vulnerability network. If a white matter tract overlaps with mostly negative edges of the vulnerability network, then its FA will likely have a significantly negative correlation with the genetic canonical score (and vice versa for positive edges). If however a white matter tract does not overlap with edges of the vulnerability network or overlaps with both positive and negative edges, then it will likely not be significantly associated with the genetic canonical scores.
3. We then calculated the genetic canonical score for each of the 20,827 individuals of the UK Biobank data.
4. Finally, we calculated the correlation in the UK Biobank data between the genetic canonical score and each IDP that were predicted to be associated with it in (2). As in [15], IDPs were adjusted for age, sex, head motion, position of the head relative to the radio-frequency coil, drift confounds, and head size (see details in [15]). We used Bonferroni correction to account for the multiple predictions made.
5. Since UK Biobank contains both participants who reported a psychiatric diagnosis and participants who did not report any (see Supplementary Table 7), this dataset also offers the opportunity to further elucidate the relationship between the white matter tracts that overlap with the vulnerability network and the presence of psychiatric disorders. We compared the values of the FA of the white matter tracts identified in (2) between people who reported a psychiatric diagnosis (‘patients’) and people who did not report any (‘controls’).

**Supplementary Fig. 1** Flowchart for the selection of SNPs and proxies which form the genomic input to the CCA analysis.


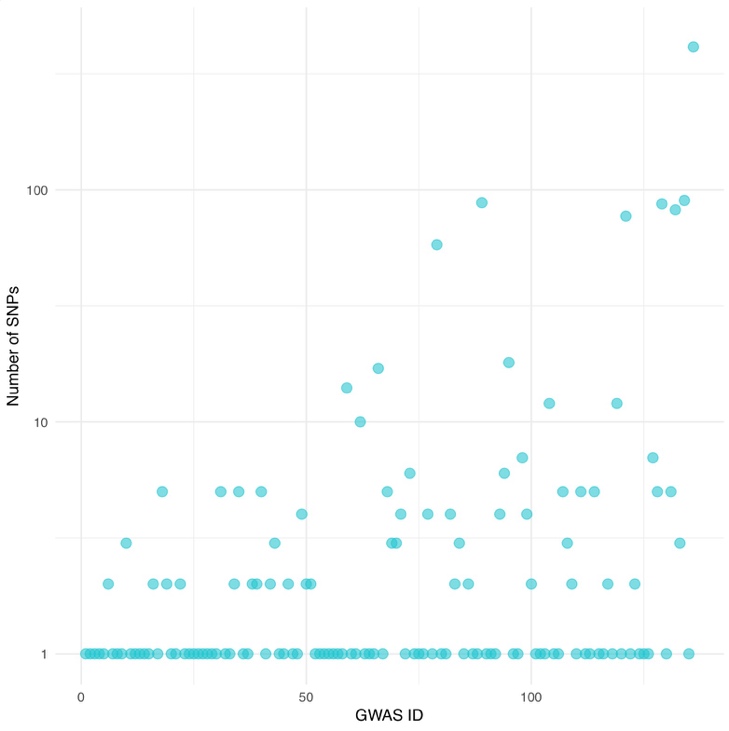


**Supplementary Fig. 2** Distribution of the number of SNPs identified by each GWAS. 58% of GWAS identified a single SNP, the majority of the others identified between 2 and 100 SNPs and one GWAS (focused on educational attainment) identified over 100 SNPs.

**Supplementary Fig. 3** P-values (blue curves) and corresponding R^2^ (red curves) for the association between PRS and the connectomic canonical score, as a function of P-thresholds for different phenotypes. The maximum P-threshold explored in each case is that which was reported in the original GWAS. Note that in PRSice, if several P-thresholds lead to the same SNPs being included, the PRS is only calculated once for all these thresholds (as all these thresholds would lead to the same PRS). For instance, this explains why only a few PRS are calculated for the cross-disorder phenotype: only a single SNP is included at a threshold of 0.022 and this remains so up to a threshold of 0.041 at which point two SNPs are included, then a third SNP is included at a threshold of 0.06, and so on.


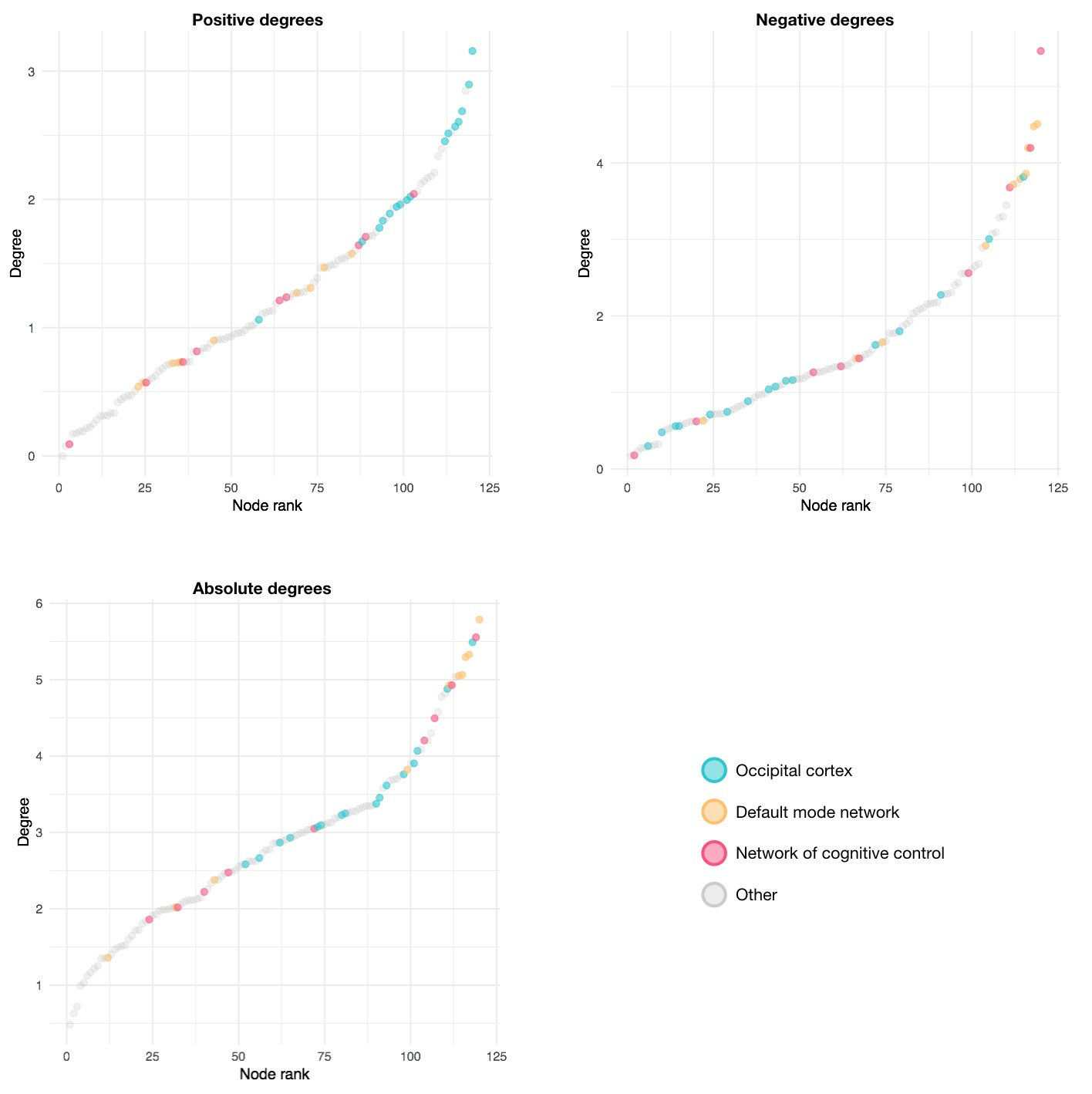


**Supplementary Fig. 4** Distribution of the degrees of nodes showing that hubs of the network overlap with three system: the occipital cortex (mostly in positive degrees), the default mode network (mostly in negative and absolute degrees), and a network of cognitive control (mostly in negative and absolute degrees).

**Supplementary Fig. 5** P-values of all correlations between the canonical brain network and the social, emotional, and behavioral variables. These p-values were the input to the combined probability test. The skewness of the distribution towards the low p-values indicate a globally positive correlation between behaviors related to vulnerability to psychiatric illness and the vulnerability network (which is further quantitatively demonstrated by the permutation-based combined probability test in the main manuscript). The absence of p-values above 0.95 indicates that no variable related to an increased vulnerability to psychiatric illness is significantly *negatively* correlated with the brain vulnerability network.


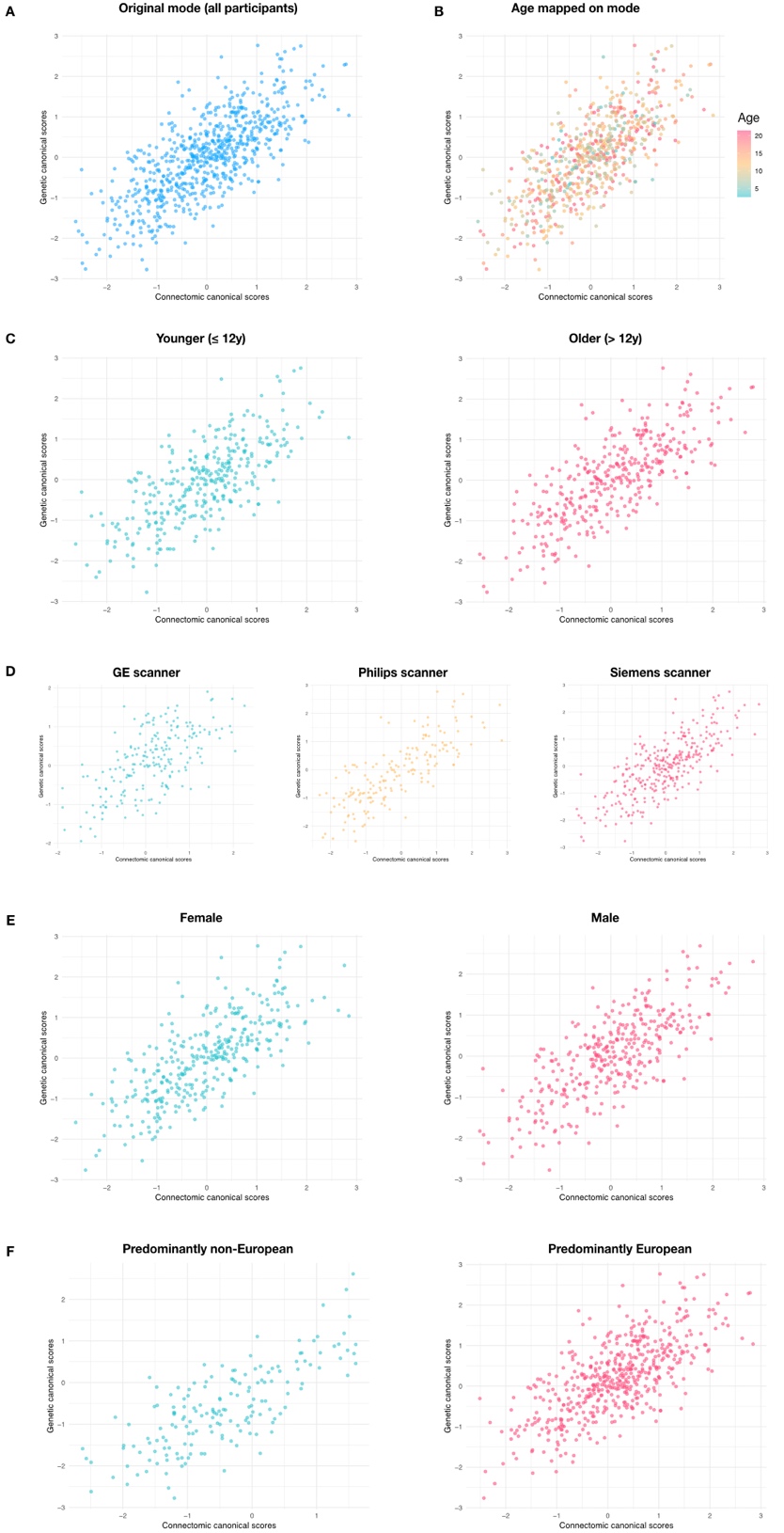


**Supplementary Fig. 6** Breakdown of the mode of population covariation by age, scanner, sex, and genetic ancestry factor. (A) Original mode of covariation showing the correlation between the connectomic canonical scores and the genetic canonical scores. Each dot is a participant. (B) Age mapped onto the mode of covariation showing that age is adjusted for in the analysis as demonstrated by the absence of an association between age of individual and their position on the mode of population covariation. (C) Breakdown of the mode of population covariation by age group. (D) Breakdown by scanner manufacturer. (E) Breakdown by sex. (E) Breakdown by ancestry showing that both participants who are predominantly of European ancestry and those who are predominantly of non-European ancestry present the same covariation pattern.

**Supplementary Fig. 7** Results of the robustness analysis with respect to the number of principal components (PC) kept in the preprocessing of the genomic and connectomic data. All correlations were positive and statistically significant at p<0.001 demonstrating robustness of the analysis to the choice of the number of PC in the preprocessing of the data.

**Supplementary Fig. 8** Three imaging-derived phenotypes (IDPs) were predicted to be associated with the genetic canonical score in UK Biobank (under the assumption that the mode of genome-connectome covariation remains unchanged in adulthood): the FA of the forceps major (left), the left acoustic radiation (middle), and the right acoustic radiation (right).

**Supplementary Table 1** PING sample demographics (N=678)

|  | Mean (s.d.; range) or *N* (%) |
| --- | --- |
| **Whole sample** | |
| Sex |  |
| Female | 336 (49.6%) |
| Male | 342 (50.4%) |
| Age (at imaging) | 12.8 (4.9; 3.2–21) |
| Genetic ancestry factors |  |
| African | 0.13 (0.27; 0–1.00) |
| American Indian | 0.04 (0.10; 0–0.83) |
| Central Asian | 0.03 (0.14; 0–1.00) |
| East Asian | 0.08 (0.24; 0–1.00) |
| Europe | 0.72 (0.36; 0–1.00) |
| Oceanic | 0.001 (0.006; 0–0.05) |
| **Sample with social, emotional, and behavioral data (PhnX questionnaire)** | |
| Sex |  |
| Female | 58 (49.6%) |
| Male | 59 (50.4%) |
| Age (at PhnX questionnaire) | 15.8 (4.1; 9.2–22.6) |
| Genetic ancestry factors |  |
| African | 0.05 (0.17; 0–0.98) |
| American Indian | 0.04 (0.09; 0–0.46) |
| Central Asian | 0.03 (0.13; 0–1.00) |
| East Asian | 0.11 (0.30; 0–1.00) |
| Europe | 0.77 (0.35; 0–1.00) |
| Oceanic | 0.002 (0.008; 0–0.05) |

**Supplementary Table 2** Phenotypes selected for the analysis and their assigned category. The names are as they appear in the NHGRI-EBI GWAS Catalog. B = Brain structure/function, CB = Cognition/Behavior, N = Neurological, Pa = Psychiatry (addiction subgroup), Pm = Psychiatry (mood disorders subgroup), Pp = Psychiatry (psychosis and autism subgroup), Po = Psychiatry (others).

| **Phenotype** | **Category** |
| --- | --- |
| Brain measurement | B |
| Infant head circumference | B |
| Hippocampal volume | B |
| Intracranial volume measurement | B |
| Hippocampal atrophy | B |
| Cortical thickness | B |
| Brain connectivity measurement | B |
| White matter hyperintensity measurement | B |
| Putamen volume | B |
| Caudate nucleus volume | B |
| Facial width measurement | B |
| Cerebral blood flow measurement | B |
| Volumetric brain MRI | B |
| Schizophrenia, mean fractional anisotropy measurement, white matter microstructure measurement | B |
| White matter microstructure (global fractional anisotropy) | B |
| Superior frontal gyrus grey matter volume | B |
| Brain volume in infants (grey matter) | B |
| Neuroimaging measurement, South Texas Assessment of Neurocognition | B |
| Reasoning | CB |
| Information processing speed | CB |
| Reproductive behaviour measurement | CB |
| Chronotype measurement | CB |
| Cognitive function measurement | CB |
| Sleep measurement | CB |
| Mathematical ability | CB |
| Reading | CB |
| Cognition | CB |
| Intelligence | CB |
| Intelligence, self reported educational attainment | CB |
| Mental process | CB |
| Aggressive behavior | CB |
| Narcolepsy with cataplexy | N |
| Functional laterality, dyslexia | N |
| Sleep duration | N |
| Migraine without aura | N |
| Sleep duration, circadian rhythm, excessive daytime sleepiness measurement, insomnia measurement | N |
| Tourette syndrome | N |
| Language impairment | N |
| Sensory perception of sound | N |
| Generalised epilepsy | N |
| Multiple sclerosis | N |
| Intracranial aneurysm | N |
| Migraine | N |
| Migraine without aura | N |
| Migraine - clinic-based | N |
| Epilepsy | N |
| Epilepsy (generalized) | N |
| Early-onset Parkinson's disease | N |
| Nicotine dependence | Pa |
| Drinking behavior | Pa |
| Alcohol drinking | Pa |
| Alcohol and nicotine codependence | Pa |
| Alcohol dependence | Pa |
| Smoking behavior | Pa |
| Alcohol consumption measurement | Pa |
| Alcohol dependence measurement | Pa |
| Opioid dependence | Pa |
| Alcohol consumption measurement, alcohol drinking | Pa |
| Alcohol dependence, risky sexual behaviour measurement | Pa |
| Cocaine dependence | Pa |
| Longitudinal alcohol consumption measurement | Pa |
| Opioid dependence, methadone dose measurement | Pa |
| Smoking behaviour measurement | Pa |
| Response to trauma exposure, alcohol dependence measurement | Pa |
| Addiction | Pa |
| Drug abuse | Pa |
| Alcohol dependence (age at onset) | Pa |
| Panic disorder | Pm |
| Unipolar depression | Pm |
| Mood disorder | Pm |
| Neuroticism measurement | Pm |
| Unipolar depression, depressive symptom measurement | Pm |
| Mood instability measurement | Pm |
| Unipolar depression, mental or behavioural disorder, bipolar disorder | Pm |
| Unipolar depression, bipolar disorder | Pm |
| Anxiety disorder | Pm |
| Personality trait | Po |
| Attention deficit hyperactivity disorder | Po |
| Conduct disorder | Po |
| Social communication impairment | Po |
| Mental or behavioural disorder | Po |
| Attention deficit hyperactivity disorder, unipolar depression,  schizophrenia, autism spectrum disorder, bipolar disorder | Po |
| Extraversion | Po |
| Wellbeing measurement | Po |
| Behavior or behavioral disorder measurement, personality trait, bipolar disorder | Po |
| Attention deficit hyperactivity disorder, bipolar disorder | Po |
| Emotional symptom measurement | Po |
| Post-traumatic stress disorder | Po |
| Bipolar disorder | Pp |
| Autism | Pp |
| Schizophrenia | Pp |
| Schizophrenia, bipolar disorder, schizoaffective disorder | Pp |
| Schizophrenia, bipolar disorder | Pp |
| Schizophrenia, autism spectrum disorder | Pp |
| Manic episode measurement, depressive episode measurement, bipolar disorder | Pp |
| Autism spectrum disorder, behavior or behavioral disorder measurement | Pp |

Supplementary Table 3 Index SNP for each phenotype with SNPs associated with the mode of population covariation (at FDR-corrected p<0.05), sample size of the GWAS, effect size of the association, and mapped gene(s) (as reported in the GWAS catalog)

| Phenotype | Index SNP | Statistic of association  (OR or beta) | GWAS  sample size | Mapped gene(s) |
| --- | --- | --- | --- | --- |
| Educational attainment | rs166820 | 0.022 (beta) | 248482 | MIR3660 |
| Schizophrenia | rs13164092 | 1.06 (OR) | 118495 | EGR1, ETF1 |
| Neuroticism | rs1557341 | 0.021 (beta) | 170911 | CELF4 |
| Depression severity | rs62100776 | 5.66 (OR) | 549935 | DCC |
| Age at first child (how young) | rs6885307 | 0.107 (beta) | 238064 | MRPS30, HCN1 |
| Mood instability | rs10959826 | 0.06 (beta) | 113968 | PTPRD |
| Smoking behavior | rs6474412 | 0.29 (beta) | 85997 | CHRNB3, CHRNA6 |
| Intelligence | rs2490272 | 7.44 (OR) | 78308 | FOXO3, AFG1L |
| Bipolar disorder | rs12202969 | 1.12 (OR) | 24025 | POU3F2, MIR2113 |
| Panic disorder | rs12579350 | (unreported) | 400 | TMEM16B |
| Migraine without aura | rs9349379 | 1.14 (OR) | 147970 | TSC1, TBC1D7, PHACTR1 |
| Schizophrenia/Autism | rs4129585 | 1.07 (OR) | 17968 | LINC00051, TSNARE1 |
| Facial width | rs17106852 | 1.10 (OR) | 3118 | Intergenic |
| Alcohol dependence | rs115460205 | (unreported) | 3924 | FSTL5 |
| Nicotine dependence | rs1051730 | 0.1 (beta) | 15843 | CHRNA5, CHRNA3, CHRNB4 |
| Narcolepsy with cataplexy | rs10995245 | 1.23 (OR) | 17374 | ZNF365 |
| White matter hyperintensity | rs3744028 | 0.12 (beta) | 12385 | TRIM65 |

Supplementary Table 4 Results from the analysis of polygenic risk scores (PRS). The P-threshold corresponds to the best P-threshold identified by PRSice-2 (i.e. the one that maximizes R^2^), R^2^ is the corresponding R^2^, p is the uncorrected p-value, and the corrected p-value is the one obtained using a permutation test and correction for multiple comparisons across the different P-thresholds.

| **Phenotype** | **P-threshold** | **R^2^** | **p** | **p (corrected)** |
| --- | --- | --- | --- | --- |
| Bipolar disorder-Schizophrenia | 0.0340 | 2.14% | 4.8E-05 | 0.0010 |
| Eating disorder | 0.0268 | 0.24% | 0.1734 | 0.7922 |
| Major depressive disorder | 0.0002 | 1.18% | 0.0026 | 0.0160 |
| Bipolar disorder | 0.0226 | 1.05% | 0.0045 | 0.0679 |
| Schizophrenia | 0.0175 | 1.07% | 0.0042 | 0.0300 |
| ADHD | 0.0914 | 0.21% | 0.2054 | 0.8202 |
| Alcohol use disorder | 0.0097 | 1.44% | 0.0009 | 0.0230 |
| Autism spectrum disorders | 0.0251 | 0.70% | 0.0203 | 0.2058 |
| Cross-disorder | 0.1129 | 0.85% | 0.0106 | 0.0300 |
| Educational attainment | 0.0148 | 1.03% | 0.0051 | 0.0290 |

Supplementary Table 5 Hubs of the network of vulnerability to psychiatric illness. Positive (negative) hubs are highly positively (negatively) connected with other brain regions. In other words, they have the highest degree centrality when considering positive (negative) connections only. Absolute hubs are highly connected with other brain regions in absolute value. In other words, they have the highest degree centrality when considering the absolute value of connections.

| **Positive hubs** | **Negative hubs** | **Absolute hubs** |
| --- | --- | --- |
| Right Superior occipital gyrus | Left Middle cingulate gyrus | Right Angular gyrus |
| Left Lingual gyrus | Right Supramarginal gyrus | Left Middle cingulate gyrus |
| Right Occipital fusiform gyrus | Left Anterior cingulate gyrus | Right Middle occipital gyrus |
| Left Superior occipital gyrus | Right Angular gyrus | Left Posterior cingulate gyrus |
| Left Frontal operculum | Left Planum temporale | Left Angular gyrus |
| Right Supplementary motor cortex | Right Subcallosal area | Left Supramarginal gyrus |
| Right Lingual gyrus | Right Middle cingulate gyrus | Right Supramarginal gyrus |
| Left Superior parietal lobule | Left Supramarginal gyrus | Left Middle temporal gyrus |
| Left Occipital fusiform gyrus | Right Parietal operculum | Left Anterior cingulate gyrus |
| Left Postcentral gyrus | Left Middle temporal gyrus | Left Inferior occipital gyrus |

Supplementary Table 6 Statistical tests for the comparison between the degrees of the nodes in three brain systems compared to other nodes in the network.

|  | **Occipital cortex** | **Default mode network** | **Network of cognitive control** |
| --- | --- | --- | --- |
| **Positive**  **Degrees** | Mean: 2.19 vs 1.06  95% C.I. of diff: [0.80 1.45]  t=6.82  **p = 3.8 x 10^-10^** | Mean: 0.98 vs 1.24  95% C.I. of diff: [-0.74 0.22]  t=-1.07  p = 0.29 | Mean: 1.12 vs 1.24  95% C.I. of diff: [-0.64 0.39]  t=-0.48  p = 0.63 |
| **Negative**  **Degrees** | Mean: 1.32 vs 1.71  95% C.I. of diff: [-0.97 0.21]  t=-1.28  p = 0.20 | Mean: 3.12 vs 1.46  95% C.I. of diff: [1.06 2.27]  t=5.47  **p = 2.8 x 10^-7^** | Mean: 2.31 vs 1.46  95% C.I. of diff: [0.19 1.51]  t=2.54  **p = 0.012** |
| **Absolute**  **Degrees** | Mean: 3.51 vs 2.77  95% C.I. of diff: [0.14 1.36]  t=2.42  **p = 0.017** | Mean: 4.10 vs 2.70  95% C.I. of diff: [0.69 2.12]  t=3.92  **p = 1.5 x 10^-4^** | Mean: 3.42 vs 2.70  95% C.I. of diff: [-0.001 1.45]  t=1.98  p = 0.050 |

**Supplementary Table 7** Diagnosis distribution in UK Biobank

|  | Number |
| --- | --- |
| **No self-reported psychiatric diagnosis** | **17,321** |
| **Any self-reported psychiatric diagnosis** | **3,506** |
| Anxiety/Panic attacks | 912 |
| Mixed anxiety and depressive disorder | 56 |
| Panic disorder [episodic paroxysmal anxiety] | 18 |
| Obsessive compulsive disorder | 13 |
| Specific (isolated) phobia | 22 |
| Nervous breakdown | 48 |
| Anxiety disorder, unspecified | 212 |
| Depression | 2,397 |
| Depressive episode | 464 |
| Deliberate self-harm/suicide attempt | 12 |
| Bipolar disorder/mania | 74 |
| Acute intoxication (alcohol) | 49 |
| Withdrawal state (alcohol) | 9 |
| Harmful substance use (alcohol) | 74 |
| Dependence syndrome (alcohol) | 30 |
| Harmful use (tobacco) | 330 |
| Dependence syndrome (tobacco) | 11 |
| Psychological/psychiatric problem | 19 |

**References**

1. MacArthur J, et al. (2017) The new NHGRI-EBI Catalog of published genome-wide association studies (GWAS Catalog). *Nucleic Acids Res* 45(D1):D896–D901.

2. Johnson AD, et al. (2008) SNAP: a web-based tool for identification and annotation of proxy SNPs using HapMap. *Bioinformatics* 24(24):2938–2939.

3. Alexander DH, Novembre J, Lange K (2009) Fast model-based estimation of ancestry in unrelated individuals. *Genome Res* 19(9):1655–1664.

4. Machiela MJ, Chanock SJ (2015) LDlink: a web-based application for exploring population-specific haplotype structure and linking correlated alleles of possible functional variants. *Bioinformatics* 31(21):3555–3557.

5. White N, Roddey C, Shankaranarayanan A, Han E, Rettmann D, Santos J, et al. (2010): PROMO: Real-time prospective motion correction in MRI using image-based tracking. Magn Reson Med. 63: 91–105.

6. Andersson JLR, Skare S, Ashburner J (2003): How to correct susceptibility distortions in spin-echo echo-planar images: application to diffusion tensor imaging. *Neuroimage*. 20: 870–888.

7. Grau V, Mewes AUJ, Alcañiz M, Kikinis R, Warfield SK (2004): Improved watershed transform for medical image segmentation using prior information. *IEEE Trans Med Imaging*. 23: 447–458.

8. Akhondi-Asl A, Warfield SK (2013): Simultaneous truth and performance level estimation through fusion of probabilistic segmentations. *IEEE Trans Med Imaging*. 32: 1840–1852.

9. Velasco-Annis C, Akhondi-Asl A, Stamm A, Warfield SK (2018): Reproducibility of Brain MRI Segmentation Algorithms: Empirical Comparison of Local MAP PSTAPLE, FreeSurfer, and FSL-FIRST. *J Neuroimaging*. 28: 162–172.

10. Fonov V, Evans AC, Botteron K, Almli CR, McKinstry RC, Collins DL, Brain Development Cooperative Group (2011): Unbiased average age-appropriate atlases for pediatric studies. *Neuroimage*. 54: 313–327.

11. Taquet M, Scherrer B, Boumal N, Peters JM, Macq B, Warfield SK (2015): Improved fidelity of brain microstructure mapping from single-shell diffusion MRI. *Med Image Anal*. 26: 268–286.

12. Crossley NA, Mechelli A, Scott J, Carletti F, Fox PT, McGuire P, et al. The hubs of the human connectome are generally implicated in the anatomy of brain disorders. Brain. 2014;137:2382–2395.

13. Greicius MD, Krasnow B, Reiss AL, Menon V. Functional connectivity in the resting brain: a network analysis of the default mode hypothesis. Proc Natl Acad Sci U S A. 2003;100:253–258.

14. McTeague LM, Huemer J, Carreon DM, Jiang Y, Eickhoff SB, Etkin A. Identification of Common Neural Circuit Disruptions in Cognitive Control Across Psychiatric Disorders. Am J Psychiatry. 2017;174:676–685.

15. Elliott LT, Sharp K, Alfaro-Almagro F, Shi S, Miller KL, Douaud G, et al. Genome-wide association studies of brain imaging phenotypes in UK Biobank. Nature. 2018;562:210–216.
